# Supplementary material for: Lipidic folding pathway of α-Synuclein via a toxic oligomer
Source: Nat Commun. 2025 Jan 17;16:760. doi: 10.1038/s41467-025-55849-3 (PMC11742675; doi:10.1038/s41467-025-55849-3)
Supplement: Supplementary file 1 — Supplementary Information [file 41467_2025_55849_MOESM1_ESM.pdf]

Supplementary materials for

Lipidic folding pathway of α-Synuclein via a toxic oligomer

Vrinda Sant, Dirk Matthes, Hisham Mazal, Leif Antonschmidt, Franz Wieser, Kumar T. Movellan, Kai Xue, Evgeny Nimerovsky, Marianna Stampolaki, Magdeline Nathan, Dietmar Riedel, Stefan Becker, Vahid Sandoghdar, Bert L. de Groot*, Christian Griesinger*, Loren B. Andreas*

*Corresponding authors. Email: bgroot@mpinat.mpg.de, land@mpinat.mpg.de, cigr@mpinat.mpg.de

The file includes:

Supplementary Figures 1 to 16
Supplementary Note 1
Tables S1 to S4
Supplementary References

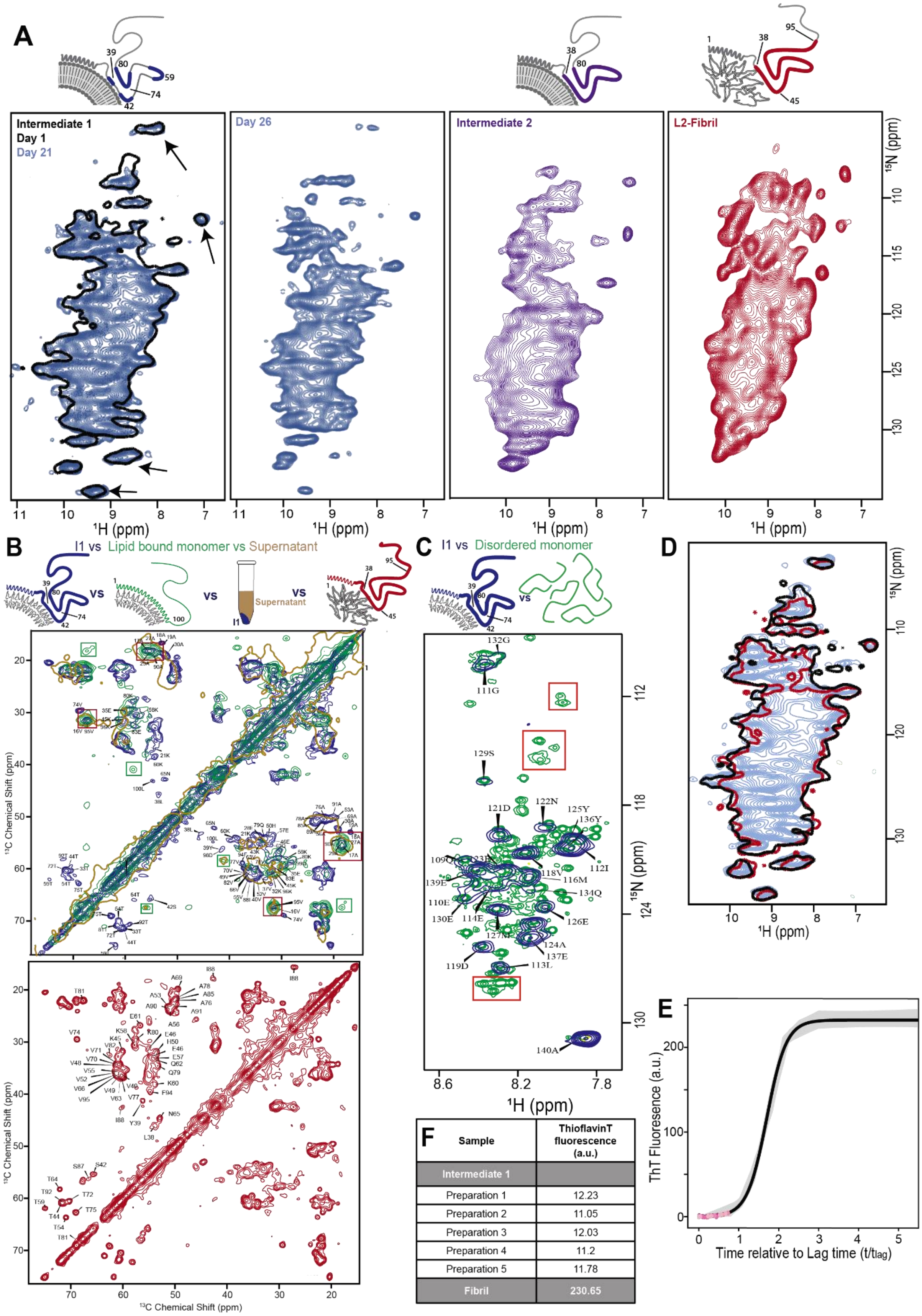

F Sample	ThioflavinT fluorescence (a.u.)
Intermediate 1	
Preparation 1	12.23
Preparation 2	11.05
Preparation 3	12.03
Preparation 4	11.2
Preparation 5	11.78
Fibril	230.65

Supplementary Figure.1: **The composition of an I1 sample and its stability.** (**A**) Stability of Intermediate 1 (I1) during measurements: (H)NH spectrum of for ^{13}C, ^{15}N- labelled αS intermediate 1 at day 1 (black outline) and at day 21 (filled blue). During this time,

measurements were continuously acquired at an estimated sample temperature of 16°C. From left to right, progression from I1 to Intermediate 2 and the fibril can be seen as observed on ^{15}N-(H)NH fingerprint spectra. Arrows mark isolated resonances that broaden out when conversion begins. When the spectrum begins to look like the one at Day 26, measurements are stopped, and a new sample is prepared. (**B**) (*Top)* The I1 sample does not contain lipid bound monomers: ^{13}C-^{13}C correlation spectrum with 20 ms DARR mixing for ^{13}C, ^{15}N labelled I1 (blue) and lipid bound monomer recorded at 850 MHz at 17 kHz MAS with sequence assignments. Resonances corresponding to V16, A17, V95, K96 and Q99 (red boxes) can be found in lipid bound monomer spectra in Antonschmidt et.al.[1] and Comellas et.al.[2] as well as the BMRB entry 6968 [https://dx.doi.org/10.13018/BMR6968] for micelle bound monomer. These resonances are also observed for the same residues in I1. However, unassigned resonances in green boxes for the lipid bound monomer spectrum are unique to the monomer and are not observed for I1 indicating that any lipid bound monomer in an I1 sample is likely below the noise threshold (average signal to noise ratio 13:1). The supernatant obtained after spinning down an I1 sample is lyophilized and rehydrated and packed into a 1.3mm rotor. The ^{13}C-^{13}C correlation spectrum (light brown) obtained shows a primarily helical species that shares several resonances with the lipid bound monomer, suggesting that monomeric species are retained in the supernatant after isolating I1. (bottom) ^{13}C-^{13}C correlation spectrum with 20 ms DARR mixing for ^{13}C, ^{15}N labelled L2-fibril reported in Antonschmidt et.al. 2021[17]. (**C**) Flexible and highly mobile monomers are depleted in I1 samples: MAS NMR INEPT-(H)NH spectrum acquired at 55 kHz MAS of I1 (blue) overlayed on the ^{1}H-^{15}N HSQC of ^{15}N- αS monomer in solution at 25°C (green). I1 peaks are assigned based on comparison with free monomer in solution. Backbone assignments were done based on BMRB entries 16300, 16904, 18857. An MAS NMR INEPT-(H)NH is expected to show only highly mobile regions in the sample. Resonances that are expected for free disordered monomer (residues 1-100) are not visible in the I1 sample (salient resonances in red boxes), indicating that the sample is depleted of highly mobile monomer required for further aggregation. For I1, resonances can be assigned to residues 109-140, confirming a flexible C-terminus. Monomer is detected in the supernatant after ultracentrifugation. (**D**) Batch-to-batch reproducibility of I1 samples. Three different samples shown in red, black and blue at day 1 of measurement have remarkable similarity. (E) Aggregation kinetics reported by ThT fluorescence. A fit from four independent repeats is shown as a solid black line. The data were fitted as described in Antonschmidt et.al.[17] using AmyloFit. The standard deviation from four samples is shown as the shaded gray area. The time axis is shown relative to the lag time, previously determined to be 6.6±2.0 hours. Data points

for ThT fluorescence for a few I1 samples used in this study, prior to isolation by ultracentrifugation are shown in different shades of pink. The lag time for these samples was determined by identifying the x-axis value on the fitted curve that corresponds to the ThT value at the time of isolation. (**F**) ThT values for some I1 and L2-fibril samples included in this study. Source data are provided as a Source Data file.

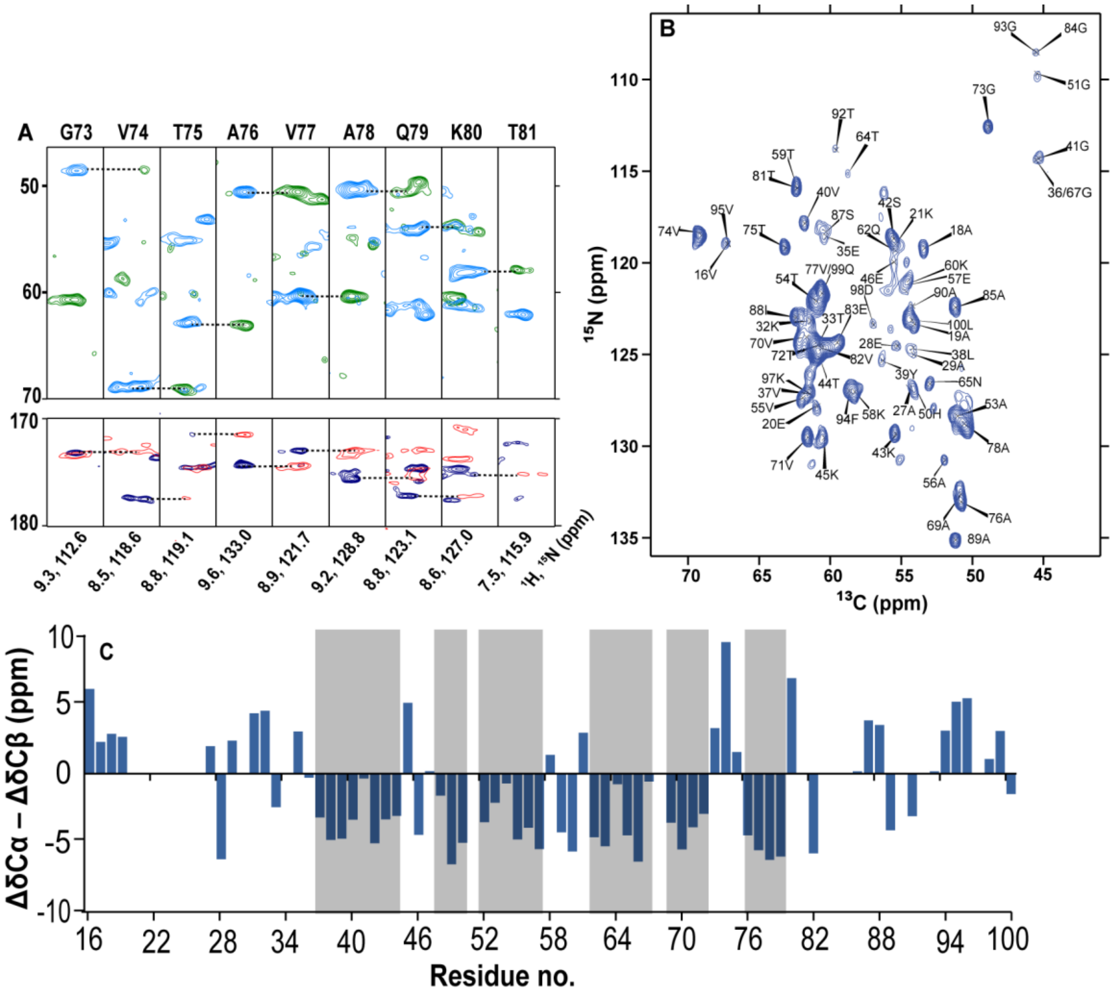

Supplementary Figure.2: **Assignments for MAS NMR spectra of I1 and its secondary structure.** (A) Sequence assignments for I1. Strip plot for backbone walk along the 73GVTAVAQKT81 stretch with proton detected spectra obtained with ^{13}C, ^{15}N-αS I1 on 800 MHz, 55 kHz MAS at an estimated sample temperature of 16°C. Blue: (H)CANH, Green: (HCO)CA(CO)NH, Red: (H)CONH, Purple: (H)CO(CA)NH. (B)2D Cα-N projection from a 3D (H)CANH spectrum with specific resonance assignments. In no case was one residue assigned to two sets of resonances indicating that the sample contains one dominant species. The average signal to noise ratio of resonances in the (H)CANH spectrum is 8.5:1. (C) Secondary structure propensity derived from Cα and Cβ chemical shifts differences from random coil shifts according to Schwarzinger et.al. by CCPN Analysis. Gray bars show stretches of β-stranded residues.

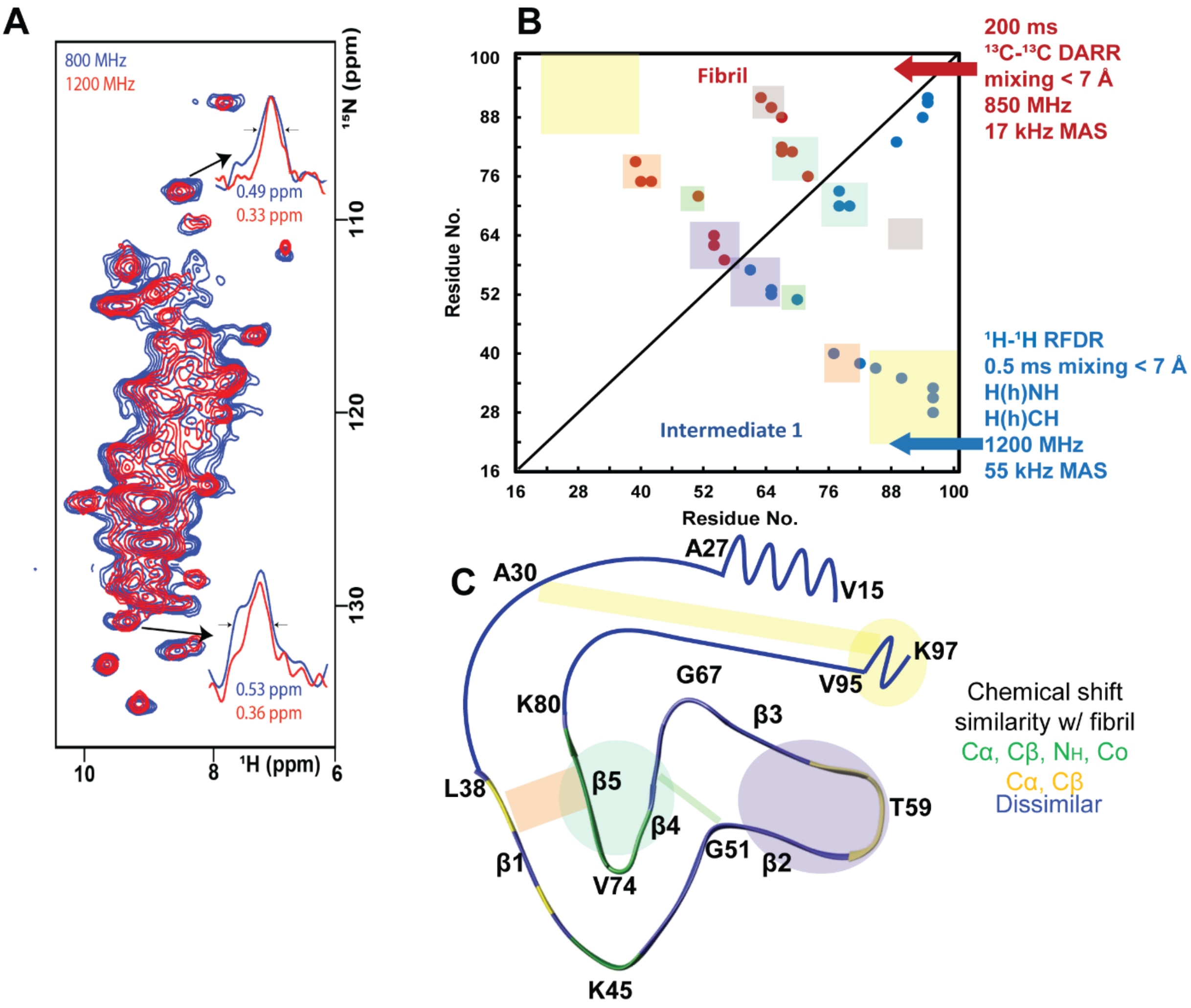

Supplementary Figure.3: **Topology of I1.** (A) Comparison of ^{1}H-^{15}N correlation spectra measured at the 1200 MHz and 800 MHz spectrometers with 55kHz MAS showing dramatic improvement in homogenous linewidths. (B) Contact map comparing long range distances (i → n (n ≥ i±4)) measured for the fibril (^{13}C-^{13}C contacts) and I1 (^{1}H-^{1}H backbone contacts). I1 contacts were recorded with (H)NHH and (H)CHH spectra. (C) Contacts mapped onto the expected fold for I1. The chemical shift similarity with the fibril is shown on the fold. As shown in Fig.1D, I1 and the L2 fibril have similar helical residues between V16-T22. Note that cryo-electron microscopy of the L2 fibril shows a β-strand for V16-T22, suggesting that a fraction of the NMR sample likely contains β-strands[3].

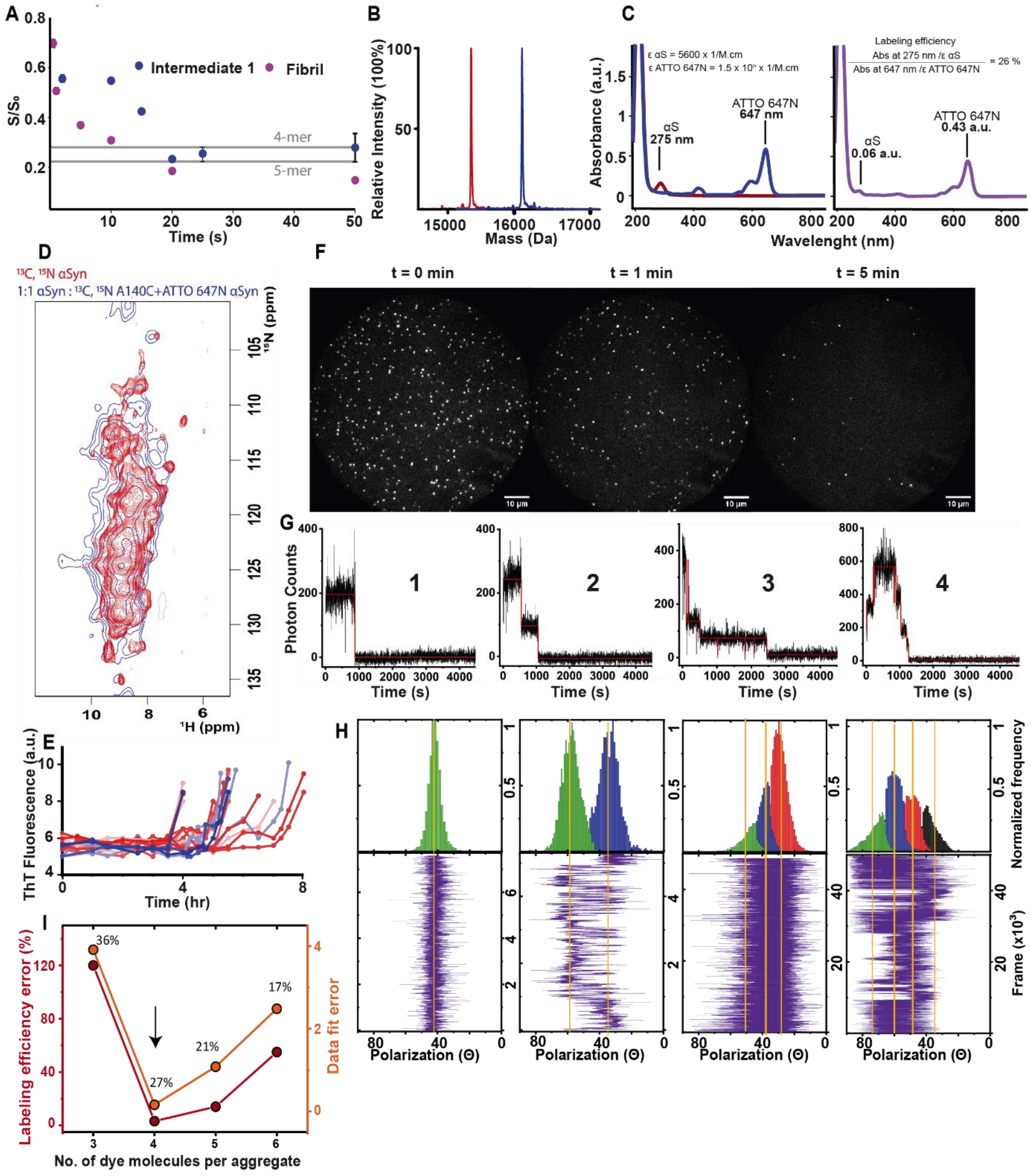

Supplementary Figure.4: Size determination of I1 by NMR and fluorescence spectroscopy. (A) CODEX curve for I1 (blue) and L2 fibril (pink) for a single ^{13}C isotopically labeled site at H50 ^{13}Cε shows that the I1 curve plateaus at ~ 0.25. Error bars are propagated from the root mean square of a noise region for each spectrum. The signal plateaus at the inverse of the number of spins over which magnetization can equilibrate and rate of decay informs about the distance between each spin. The L2 fibril curve did not fully plateau within the times used for magnetization to exchange and notably the curve decays faster suggesting a shorter distance between the isotopically labeled H50 ^{13}Cε nuclei between each molecule in the L2 fibril. Source data are provided as a Source Data file. (B) Deconvoluted mass spectra of WT

dye unlabeled αS (red) and A140C dye labeled αS (blue) showing proteins of mass 15351.72 ±2.40 Da and 16121.42±2.39 Da, respectively, confirming the dye tagging of the protein with approximately 100% yield. (C) Absorbance spectra of αS used to calculate labeling efficiency of the ATTO647N dye after mixing dye labeled protein stock with unlabeled stock. (*Left*) absorbance spectra of unlabeled αS shows a maximum at 275 nm. The extinction coefficient (ε) is stated on top and is based on tyrosine content[4]. Overlayed is the absorbance spectra of ATTO 647N in DMSO shows a maximum at 647 nm. ε stated on top is from the manufacturer. (*Right*) Absorbance spectra of about 75% unlabeled αS and 25% dye labeled αS shows a peak at 275 nm and 647 nm as expected. The concentrations are calculated according to the Beer-Lambert law using the given ε and a path length of 0.2 cm. The peak at 275 nm gives the concentration of the total protein (~54μM) and the peak at 647 nm gives the concentration of the dye labeled protein (~14 μM), resulting in a labeling efficiency of ~27%. (D) ^{1}H-^{15}N correlation spectra comparison for 1:1 dye bound: wild type. I1 wild type (red) and dye bound I1 (blue) serve as a fingerprint that confirm that an I1 type fold is retained in the dye bound aggregate. (E) ThT aggregation curves of WT dye unlabeled αS (shades of red) and A140C dye labeled αS (shades of blue) show that the kinetics of dye labeled αS are within the variation presented by dye unlabeled αS. Source data are provided as a Source Data file. (F) Snapshots of single-molecule fluorescence images at different times of I1 labeled with ATTO647N (experimental labeling efficiency of 26%). The images show dispersed aggregates and most aggregates are completely photobleached in about 5 minutes upon continuous irradiation with a laser. (G) Selected intensity time traces which show clear 1, 2, 3 and 4 bleaching steps. Source data are provided as a Source Data file. Histogram of bleaching steps is depicted in Fig.2D. (H) Examples of polarization traces (purple) with 1, 2, 3 and 4 different polarization states (yellow lines) from super-resolved images in Fig.2E-H. The precision of each localization scales as the inverse square root of the number of photons, $\sigma_{loc} \sim \frac{1}{\sqrt{N}}$, which can vary for different fluorophores due to the heterogeneity in their photophysical properties. For demonstrated time traces the average localization precision is 0.34 nm, 0.52 nm, 0.38 nm and 0.25 nm for states 1, 2, 3 and 4, respectively. The normalized segmented histograms (green, blue, red and black) represent the relative population of each polarization state in the time trace. Source data are provided as a Source Data file. (I) A binomial distribution is used to fit bleaching step histograms. Varying the total number of monomers allows one to extract the theoretical labeling efficiency as a fit parameter. The labeling error (the difference between fitted labeling efficiency and experimental value) is plotted as a function of the number of monomers per oligomer (red curve), pointing to the tetramer as the best model (black arrow). In addition, the residual of each

fit, plotted as a function of the number of monomers per oligomer (orange curve), confirms that the tetramer model fits best to the experimental data. Source data are provided as a Source Data file.

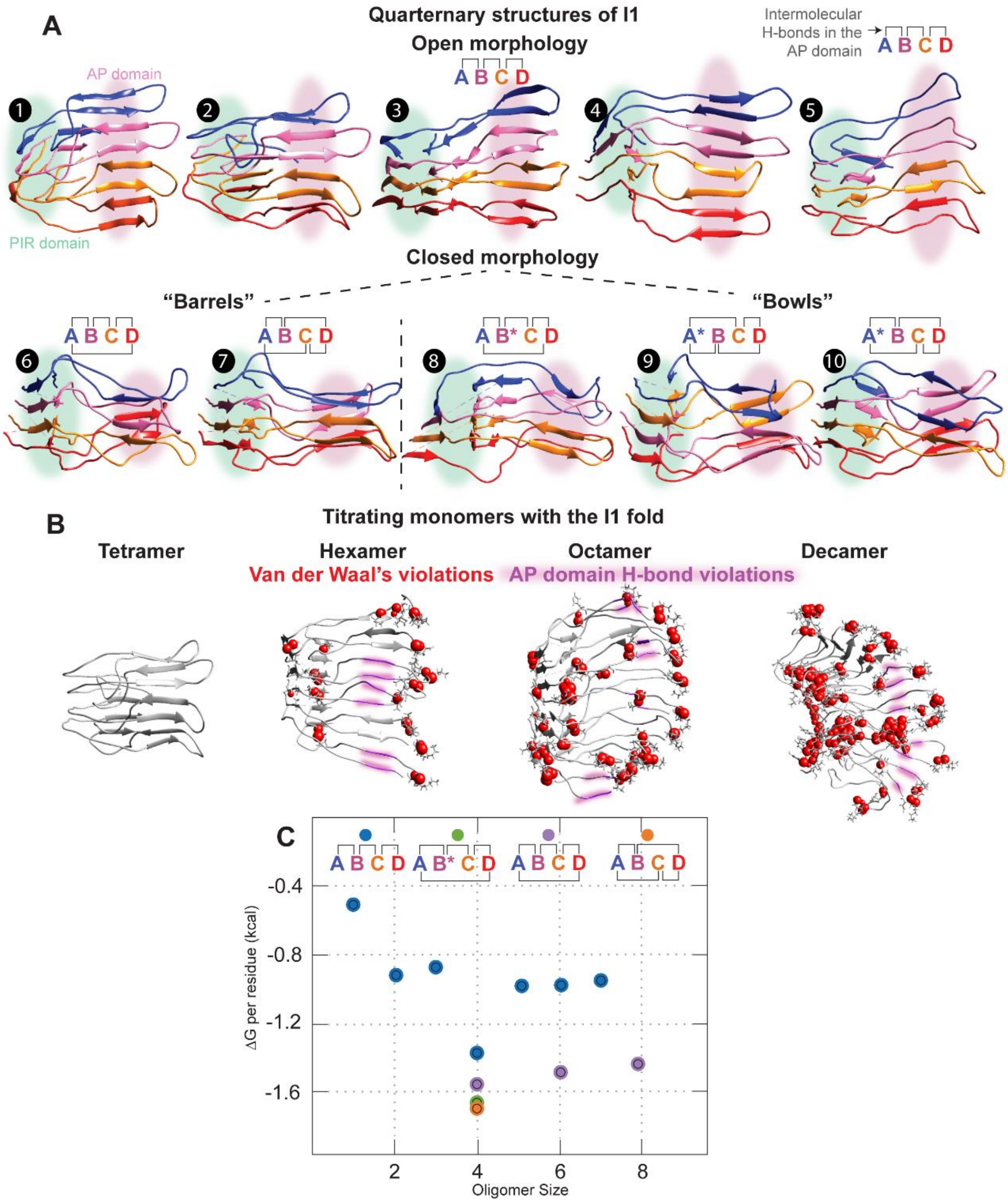

Supplementary Figure.5: Conformers of I1 (G36-K80 segment) modeled with CYANA and all-atom restrained MD show that tetramer is the optimal size for I1. NMR restraints in Supplementary Table.1 are used to construct these conformers. (A) Conformers are numbered 1-10. Two morphological categories formed by an I1-type fold: Open and closed. All

conformers feature two domains: a fibril like PIR domain and AP domain, highlighted in green and pink, respectively. Structures are colored by chain and show the pattern of intermolecular H-bonds. Chain with missing intramolecular H-bonds is denoted by a star. Open conformers differ primarily on the tightness of loops at K45 and V74. Closed structures differ in the order of intermolecular H-bonds in the AP domain. The "bowl" type sub-category of closed structures have one strand that is missing intra-molecular H-bonds. Conformers were produced with CYANA (1,2,4,5,7,9,10) and all-atom restrained MD (3,6,8). (B) Titrating monomers with the open I1 fold shows steric (red spheres) and hydrogen bond (highlighted purple ribbons) violations arising for oligomers larger than tetramers. For larger oligomers, restraints are simply replicated for each molecule and the structure calculation is performed in CYANA. Chimera is used to identify Van der Waals violations larger than 0.6 Å. H-bond violations are considered when larger than 0.3 Å. Violations are depicted if they are found in more than 15 out of 20 structures. Terminal residues are not included in the analysis. Violations occurring in the AP domain can be seen as the aggregate gets larger. (C) Solvation free energy per residue calculations[5,6] for different oligomer sizes shows that there is an energy minimum for the open and barrel morphology for the 4-mer. Different structures are denoted by chain IDs on top. Source data are provided as a Source Data file.

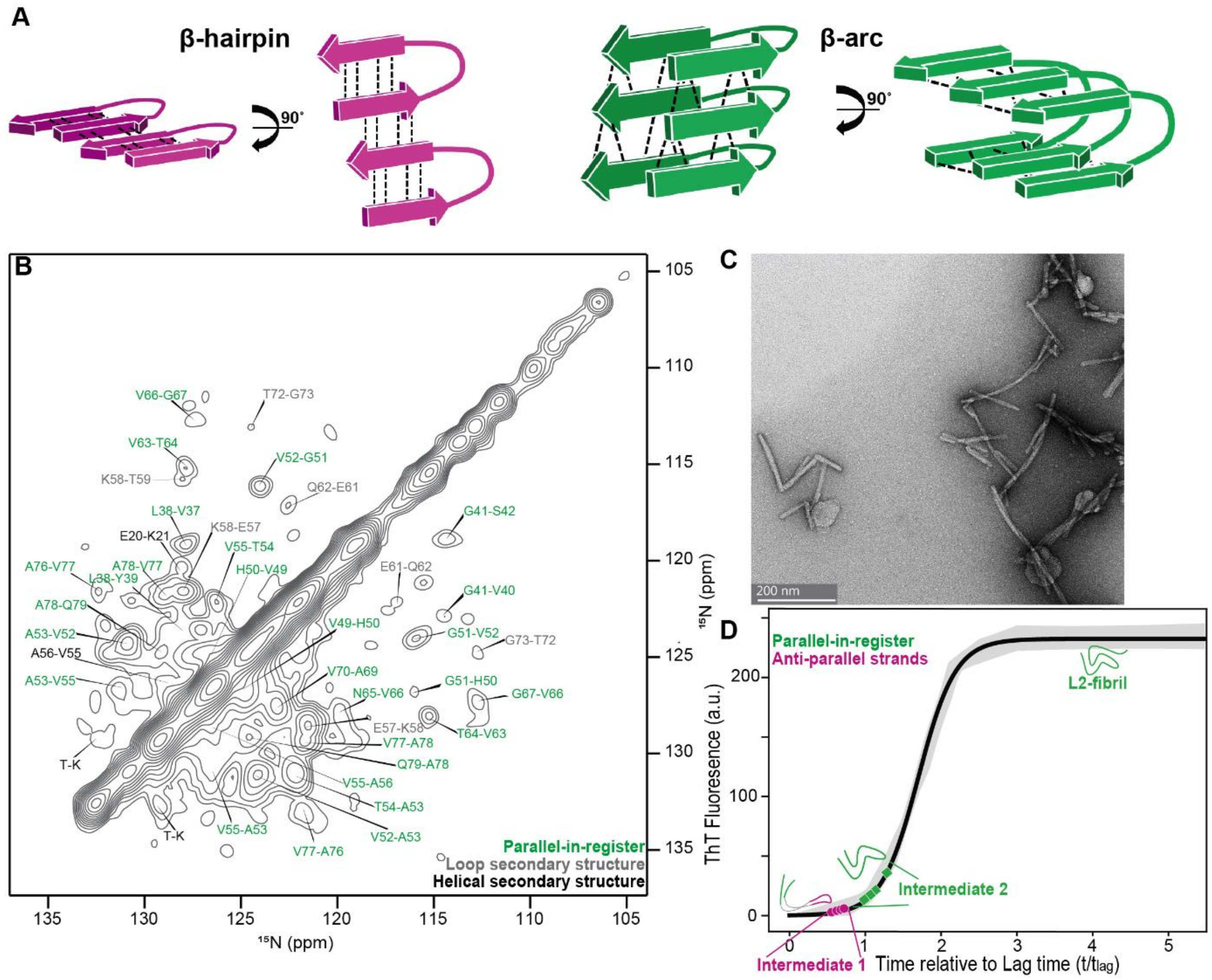

Supplementary Figure.6: Fibrillar intermediate 2 (I2) features a β-arc at V52-V66. (A) Schematic showing the distinct hydrogen bonding in a β-hairpin and β-arc. Hydrogen bonds are shown as dotted black lines. In a β-hairpin, characteristic of globular proteins, hydrogen bonding occurs between consecutive strands via backbone hydrogen bonds. In a β-arc, characteristic of amyloid fibrils, consecutive strands are held together by sidechain hydrophobic, or charge interactions. The backbone hydrogen bonds occur between consecutive molecules, leading to stacking of these molecules. **(B)** ^{15}N-^{15}N correlation spectra showing exclusively next neighbor contacts characteristic of PIR β-sheets. The I2 preparation was identified based on its characteristic (H)NH spectrum (Fig.S1A). The resonances were assigned based on chemical shift similarity to the L2 fibril and helical termini were assigned based on chemical shift similarity to I1. **(C)** TEM image of an I2 sample shows short filamentous strands representative of fibrillar intermediates. **(D)** Aggregation kinetics are observed with ThT fluorescence. The ThT values at which different samples of I1 and I2 have been isolated are shown in pink and green, respectively. I1 is primarily present in the lag phase of aggregation, whereas I2 in the growth phase. The aggregation curve is reproduced from Fig.S1E. Note that for each intermediate ThT values are shown only at the time when aggregation was stopped and samples were isolated.

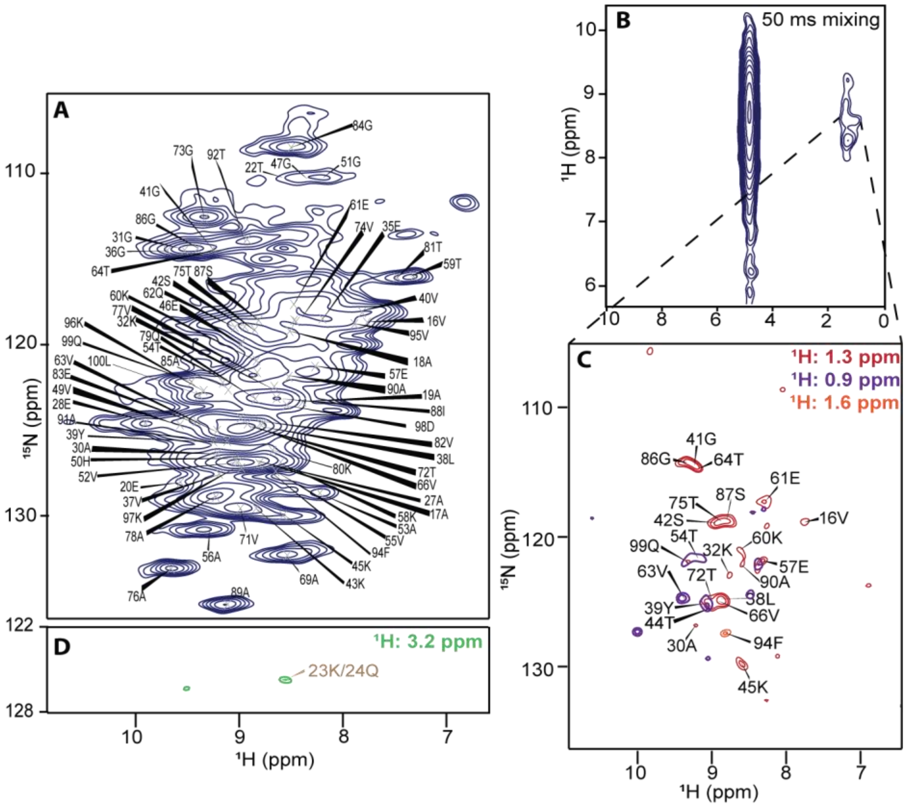

Supplementary Figure.7: Assignments of I1 spectra where magnetization has been transferred from lipid to protein. (A) An (H)NH spectrum with resonance assignments for I1. **(B)** ^{1}H-^{1}H 2D projection of a 3D H(H)NH spectrum with a z-mixing time of 50 ms. **(C)** Overlayed ^{15}N-^{1}H 2D projections of the 3D H(H)NH spectrum of planes corresponding to ^{1}H chemical shifts for lipid terminal methyls (0.9 ppm, purple), acyl chain (1.3 ppm, red) and the H_{β} on the fatty acyl chain (1.6 ppm, orange). **(D)** ^{15}N-^{1}H projections of the planes corresponding to ^{1}H chemical shifts for choline methyls (3.2 ppm, green). Tentative assignments are labeled in brown.

Supplementary Note 1

All-atom Molecular Dynamic (MD) simulations were performed in two steps to probe placement of I1 in lipid bilayers. First, a short segment of I1 (G36-T81), consisting of the four β-strands was simulated with restrained MD simulations in a total of eight different orientations on a POPC and POPA (molar ratio 1:1) lipid bilayer (Supplementary Table.3). This was followed by unrestrained MD simulations to evaluate dynamics and agreement with experimental distance restraints for orientations 1 to 4 (Fig.S8). Fig.S8 provides an overview of all simulations performed to select orientations 1 and 2 as candidates fulfilling the lipid-protein contacts and concludes that orientation 3 and those similar to it, where the AP domain is outside the bilayer, do not reproduce lipid-protein contacts. One conformer from each type of AP domain morphology (open, closed: bowl and barrel) was probed in the unrestrained simulations for orientation 1 to 4 and only the open and bowl morphologies were carried over to the next step because they showed most stable structures. In the next step, a longer segment consisting of helices and β-strands (V16-Q99) was simulated with unrestrained MD simulations. Fig.S9 shows detailed analysis of structural data that are used to assess consistency with experimentally observed parameters for the V16-Q99 segment of I1 in orientations 1, 2 and 3, in particular the agreement with experimental lipid contacts. The open and bowl morphologies in orientation 1 and 2 continue to agree with experimental lipid contacts as well as H-bonds in the AP domain.

An I1 sample thus is likely to contain an ensemble of open and bowl morphologies in orientations 1 and 2.

In the next step, MD simulations were used to probe the ability and extent of I1 in orientation 1 and 2 to permeabilize lipid bilayers at two different salt concentrations (A: 150mM NaCl; B: 100mM NaCl and 40mM $CaCl_2$). These simulations were initiated from I1 models with refined N- to C-, and C-terminal structure restraints (Supplementary Table.4).

Fig.S10 shows detailed analysis of structural data that are used to evaluate and compare with experimentally observed parameters for the V16-Q99 segment of I1 in orientations 1 and 2.

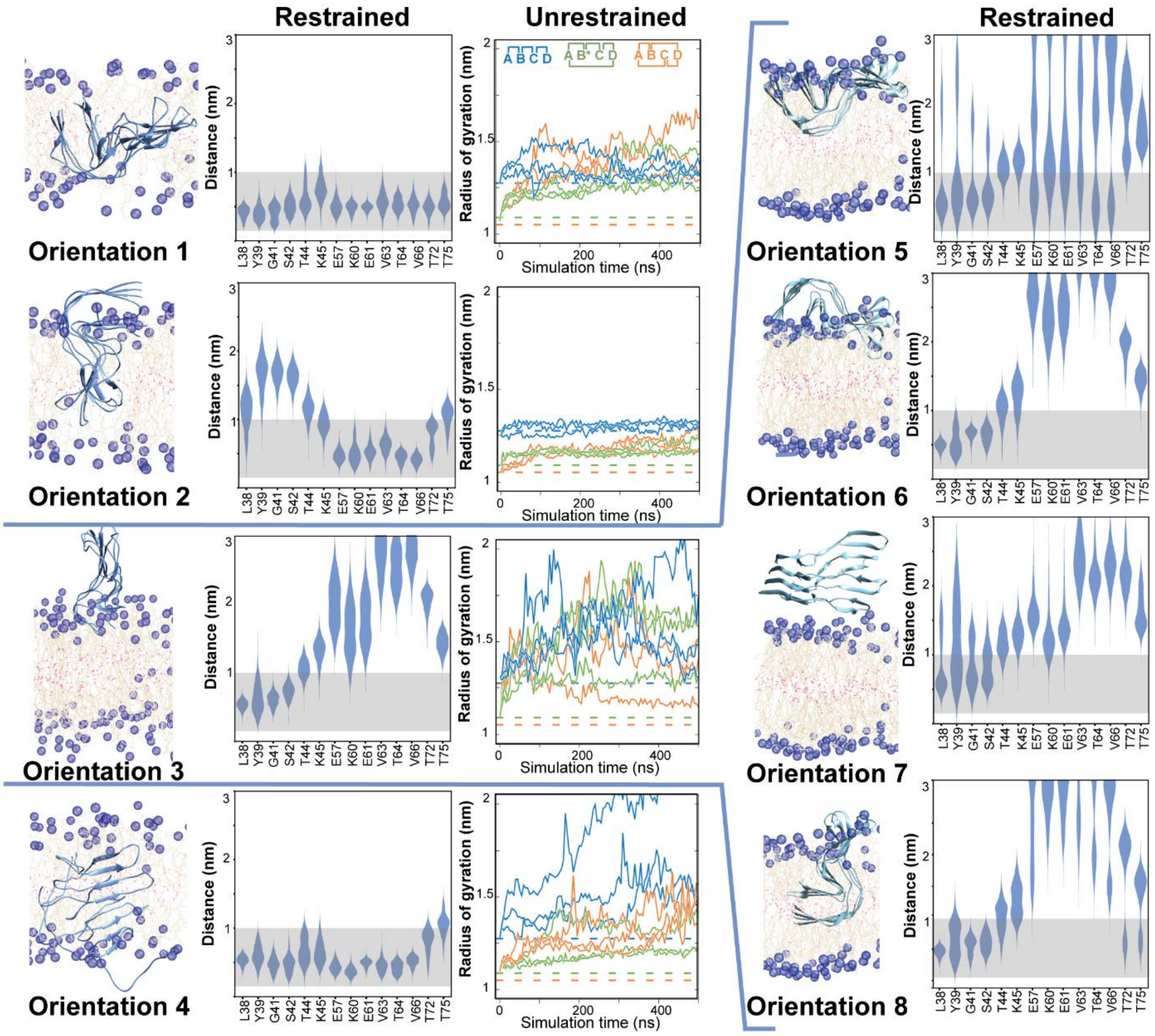

Supplementary Figure.8: Overview of initial testing which identifies orientations 1 and 2 as best candidates. To probe the orientations of I1 with respect to a bilayer, a construct (G36-T81) without the helices was used and the AP domain was restrained. Distances between lipid and protein protons during the last 250 ns of each trajectory were followed. The lipid contacts observed are under the 'restrained' column and the gray bar shows the experimental range for the lipid contacts. Here orientations 1-4 are stably bound to the bilayer whereas orientations 5-8 convert from their initial orientation (as seen in the snapshots) to the state in orientation 3 i.e. showing now close contacts to the bilayer. In this case, the AP domain hydrogen bonds behave as in orientation 3. Unrestrained simulations were performed with three morphologies (open: blue, bowl: green, barrel: orange) for orientations 1-4. Radius of gyration (backbone atoms of H50-G67) shows the AP domain dynamics compared to the initial structure (broken lines). In orientation 3 and 4, the radius of gyration shows a dynamic and expanding AP domain for all three probed morphologies. In all four cases, the barrel AP domain morphology shows dynamic structures and was not used for further analysis. Source data are provided as a Source Data MD file.

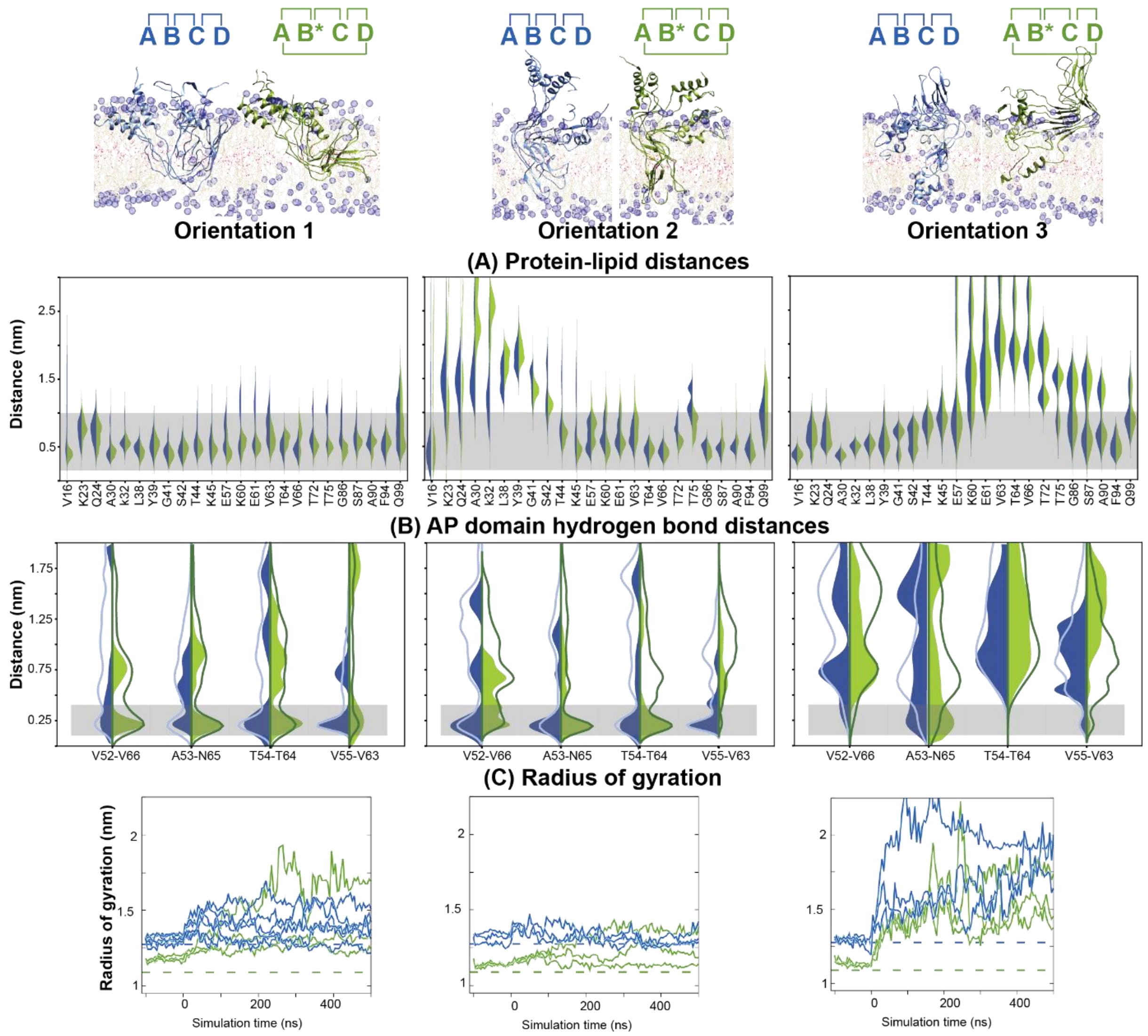

Supplementary Figure.9: MD simulations of αS I1 show that lipids are important to stabilize the AP domain. A construct V16-Q99 is simulated with two morphologies (open: blue, bowl: green). For each orientation, snapshots from the simulation are shown where purple represents headgroup choline, tan the lipid acyl chains and pink, the terminal methyl. Panels *A-D* quantify parameters from simulations for orientations 1,2, and 3 (*left to right*). Gray region in each panel shows the distance range expected from experiments and depends on the concentration of protons in the sample, mixing time and type of measurement. (A) Distances observed in the simulation between backbone amide protons and lipid protons. (B) H^N-O distances corresponding to hydrogen bonds in the AP domain. Blue and green lines show statistics accumulated from 6 simulations for orientation 1 and 3 simulations for orientations 2 and 3. Filled distributions represent statistics from the simulation that agreed best with experimental parameters. H-bond distances belonging to the same atom pair from all four different molecules were pooled together. The long H-bond distances can be attributed to the transient formation of edge strands, like in the open morphology, that have dangling inter-molecular H-bonds and the transient loss of intra-molecular H-bonds, like in the bowl morphology. Since the simulations are unrestrained, spontaneous sampling and exchange between morphologies can be observed. Simulations are considered to agree with experiment when a significant frequency can be observed within the gray range. (C) Radius of gyration (backbone atoms of H50-G67) for each scenario shows the AP domain dynamics compared to the initial structure (broken lines). Open morphology (blue traces) is rather stable in orientations

1 and 2, The bowl morphology (green traces) is more dynamic in orientation 1 compared to orientation 2. In orientation 3, both morphologies are very dynamic. Despite this, the bowl in orientation 1, satisfies H-bonds and lipid contacts for the majority of the time. Putting together the analysis of all parameters, we see that the open and bowl morphologies agree with a large fraction of experimental protein-lipid contacts and hydrogen bonds in orientations 1 and 2. In comparison to orientations 1 and 2, in orientation 3, the residues in the AP domain spend most of the time outside the bilayer. In this case, the AP domain is solvent exposed and unfolds. Fewer H-bonds are observed within the gray range and dynamics in the AP domain are observed for orientation 3. Source data are provided as a Source Data MD file.

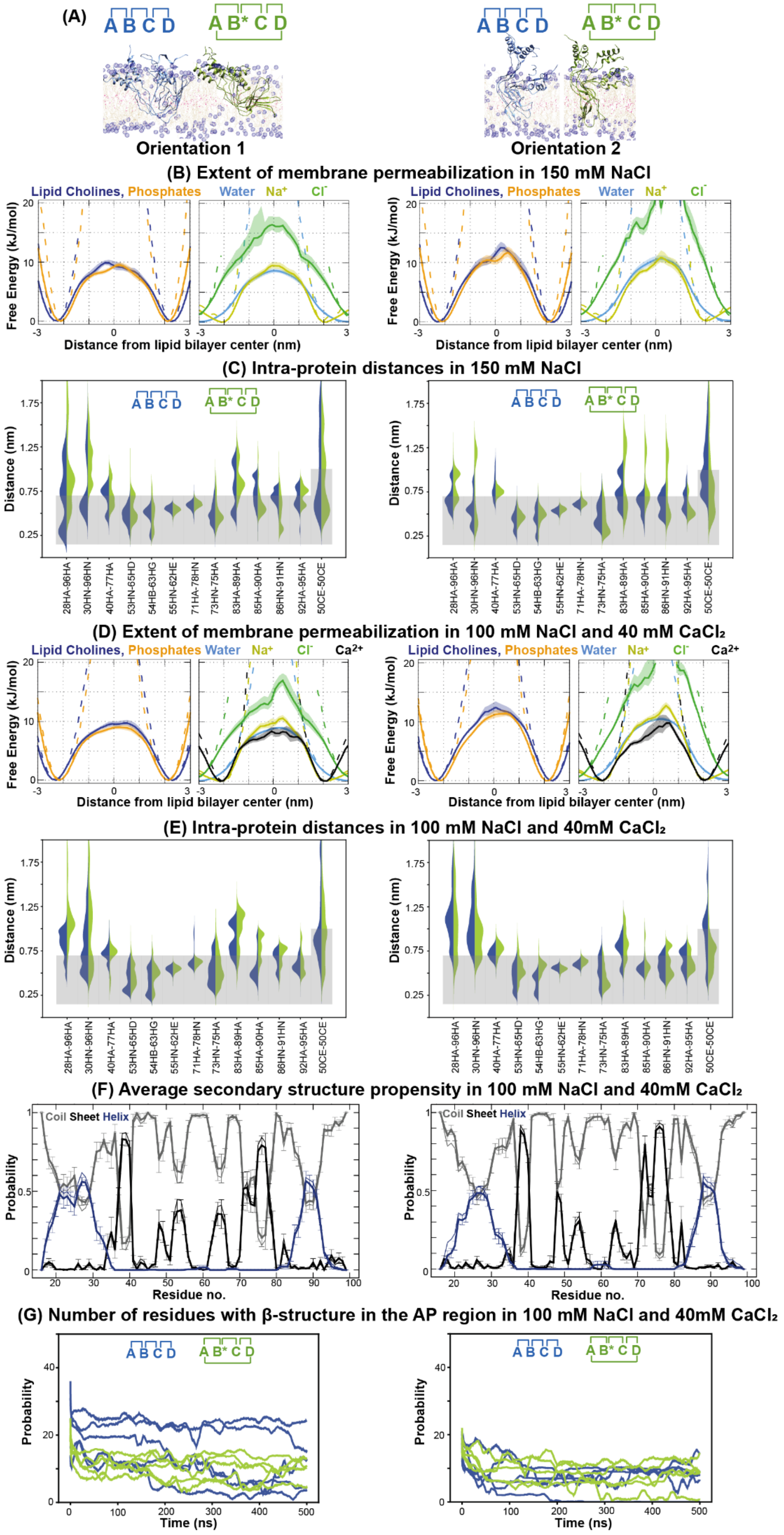

(A)
A B C D
A B* C D
Orientation 1
Orientation 2
(B) Extent of membrane permeabilization in 150 mM NaCl
Lipid Cholines, Phosphates
Water Na+ Cl-
Free Energy (kJ/mol)
Distance from lipid bilayer center (nm)
(C) Intra-protein distances in 150 mM NaCl
Distance (nm)
28HA-96HA
30HN-96HN
40HA-77HA
53HN-65HD
54HB-63HG
55HN-62HE
71HA-78HN
73HN-75HA
83HA-89HA
85HA-90HA
86HN-91HN
92HA-95HA
50CE-50CE
(D) Extent of membrane permeabilization in 100 mM NaCl and 40 mM CaCl₂
Lipid Cholines, Phosphates Water Na+ Cl- Ca2+
(E) Intra-protein distances in 100 mM NaCl and 40mM CaCl₂
(F) Average secondary structure propensity in 100 mM NaCl and 40mM CaCl₂
Coil Sheet Helix
Probability
Residue no.
(G) Number of residues with β-structure in the AP region in 100 mM NaCl and 40mM CaCl₂
Probability
Time (ns)

Supplementary Figure.10: I1 causes membrane defects in MD simulations. Bilayers equilibrated with I1 models in orientation 1 and 2 show a reduced energy barrier for permeation of lipid headgroups, water and cations across the membrane center compared to an unperturbed bilayer without bound I1. **(A)** Illustrations of orientations 1 (left) and 2 (right). The derived properties in panels B-G are presented respectively for these orientations. **(B)** Extent of membrane disruption observed in MD simulations for I1 (150mM NaCl) in orientation 1 (left) and orientation 2 (right). The density based free energy profiles across a bilayer for lipid choline (purple), phosphates (orange)**,** water (blue), Na^+ (yellow), Cl^- (green)**.** Dotted lines show the free energy profiles for unperturbed bilayers. **(C)** Intra-protein distances for open (blue) and bowl (green) morphology as observed during the simulation for orientation 1 (left) and orientation 2 (right). Gray region shows the distance range expected from experiments. **(D)** Extent of membrane disruption observed in MD simulations for I1 (100mM NaCl and 40 mM $CaCl_2$) orientation 1 (left) and orientation 2 (right). The density profiles across a bilayer for lipid choline (violet), phosphates (orange)**,** water (blue), Na^+ (yellow), Cl^- (green) and Ca^{2+} (black)**.** Dotted lines show the density profiles for unperturbed bilayers. **(E)** Intra-protein distances for open (blue) and bowl (green) morphology as observed during the simulation for orientation 1 (left) and orientation 2 (right). Gray region shows the distance range expected from experiments. **(F)** Average secondary structure propensity for I1 models in orientation 1 (left) and orientation 2 (right). **(G)** Time course of β-structure propensity for open (blue) and bowl (green) morphologies in the AP domain. The change in secondary structure in the first tens of ns is due to the removal of distance restraints used during the equilibration of the membrane inserted oligomers prior to the production runs (data not shown in the plot). After this initial phase, no significant changes in the β-structure content of the AP domain were found over time and across all probed simulation systems. Source data are provided as a Source Data MD file.

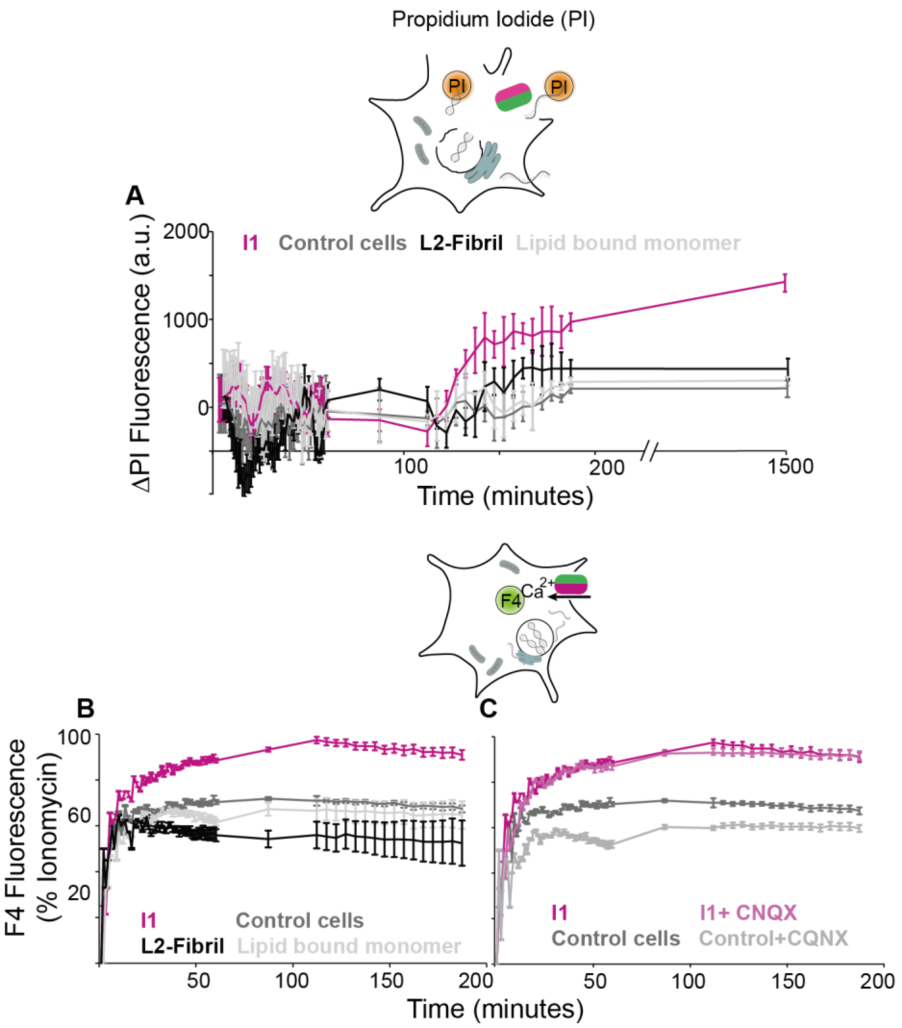

Supplementary Figure.11: I1 disrupts membranes. (A) Propidium iodide (PI) fluorescence shows that cell death begins at ~120 minutes for cells incubated with I1, whereas the Ca^{2+} influx starts to increase at ~15 minutes after incubation. PI is a cell membrane impermeable dye that fluoresces when it binds nucleic acids. Due to its impermeable nature, this happens only when the membrane ruptures as illustrated in the schematic on top. **(B)** Calcium influx is measured by fluorescence of Fluo-4 (F4) loaded in SH-SY5Y cells as in Fig.4F. The I1(pink) and control cells (dark gray) curves are reproduced from Fig.4E. Monomers (light gray) and fibrils (black) do not show a significant difference from the control. **(C)** The Ca^{2+} influx caused by I1 is not related to the action of AMPAR (AMPA glutamate receptor) as inhibiting AMPAR with CNQX (cyanquixaline) does not change the Ca^{2+} uptake curves for I1. Error bars represent standard error of the mean in panels for 6 replicates from two different preparations *(A-C)*. I1 curves in panels (B) and (C) are reproduced from Fig.4F. The concentration of αS in I1, L2-fibril and lipid bound monomer samples was 0.6 µM and the lipid concentration in each sample was estimated to be 60 µM, 3 µM and 60 µM respectively. The CNQX concentration was 5 µM. Source data are provided as a Source Data file.

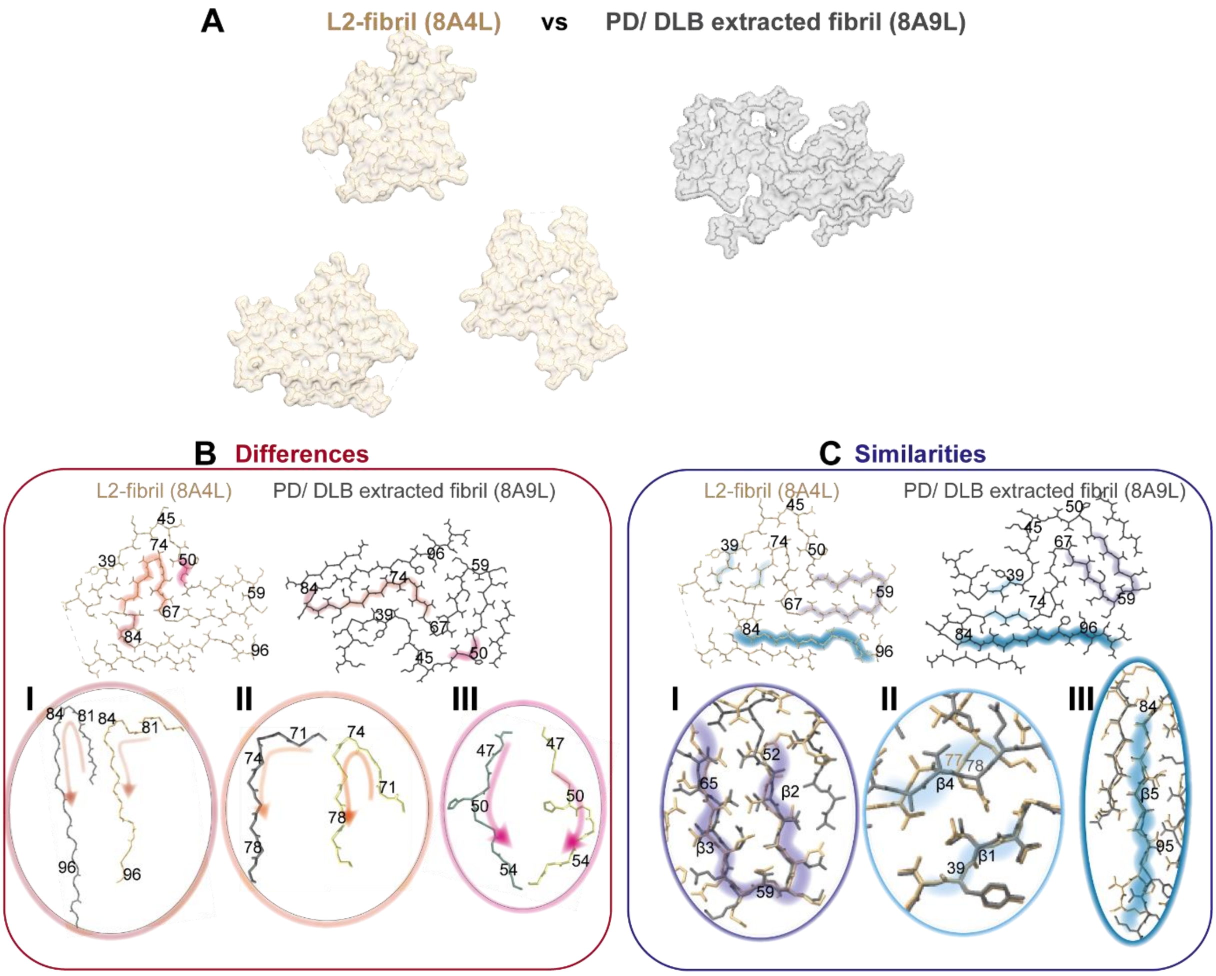

Supplementary Figure.12: Comparison of L2 fibril with the Lewy fold extracted from PD/DLB brains. (A) The L2 fibril features three proto-filaments while the PD/DLB fibrils always occur as single filaments. **(B)** Differences between the folds of individual protofilaments of the two fibrils occur at (I) the turn at G84 which is 180° in the PD/DLB fibril and 90° in the L2 fibril, (II) the at G73 which is 90° in the PD/DLB fibril and ~160° in the L2 fibril and (III) the bend at H50-G51 which is concave in the PD/DLB fibril and convex in the L2 fibril. **(C)** Differences between the folds of individual protofilaments of the two fibrils include (I) the β-arc at T59, (II) the interaction between β1 and β4 and the structure of the β5 strand.

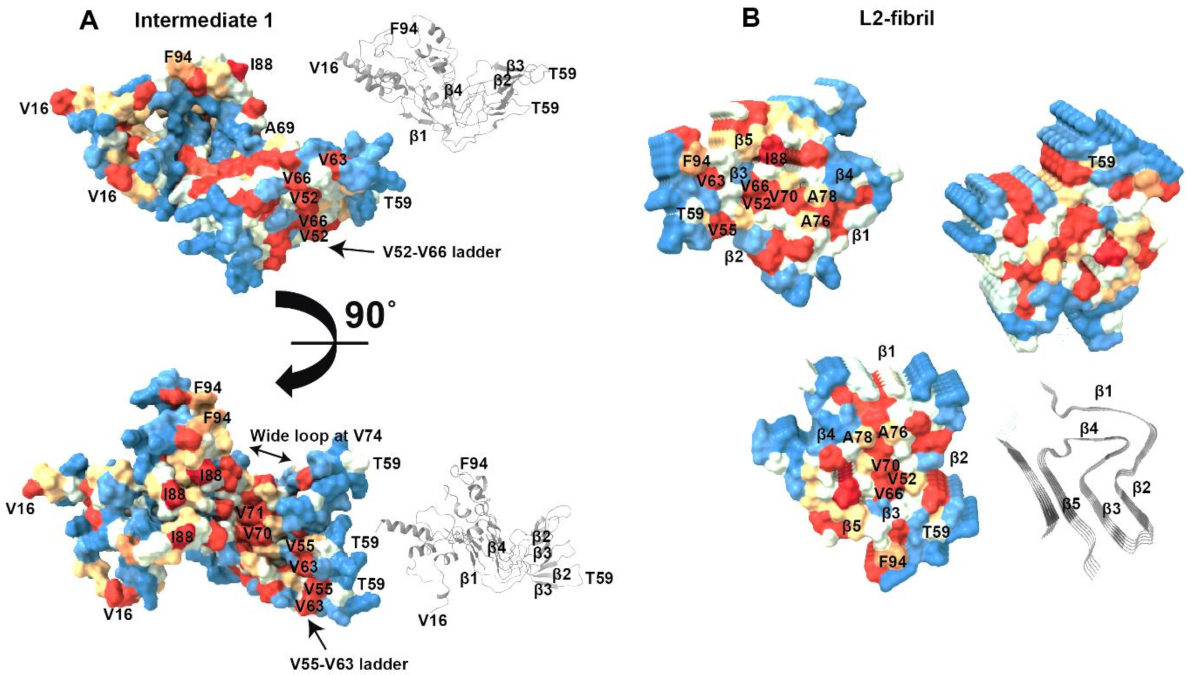

C SASA (nm²)

	I1	L2 fibril
AP	51.53 ± 1.88	35.56 ± 2.99
PIR	27.65 ± 1.90	27.12 ± 1.11

Supplementary Figure.13: Kyte-Doolittle hydrophobicity[85] surface of I1 (A) and L2-fibril (B). (A) Blue-white-red shows increasing hydrophobicity. In I1, AP β-strands (β1 and β2), have two exposed surfaces with ladders built by hydrophobic residues V52, V66 and V55, V66. Additionally, the loop at V74 is wider in I1 compared to the L2-fibril. **(B)** In the fibril, V52-V66 get buried in the hydrophobic core of the fibril, along with V71. The V74 loop gets tighter, preventing the exposure of V71 and A69 to the solvent. The hydrophobic residues F94 and I88 only get buried in the hydrophobic core of the fibril, when β5 folds in on β3. **(C)** The hydrophobic solvent accessible surface area (SASA) for the AP domain (residue 50-67) and in the PIR domain (residue 37-44 and 75-80) based on MD simulations of tetrameric models with I1- (G36-T81) and L2-fold (G36-K97). Averages (and standard deviations) were calculated for the 50 ns of three 100 ns long simulations each.

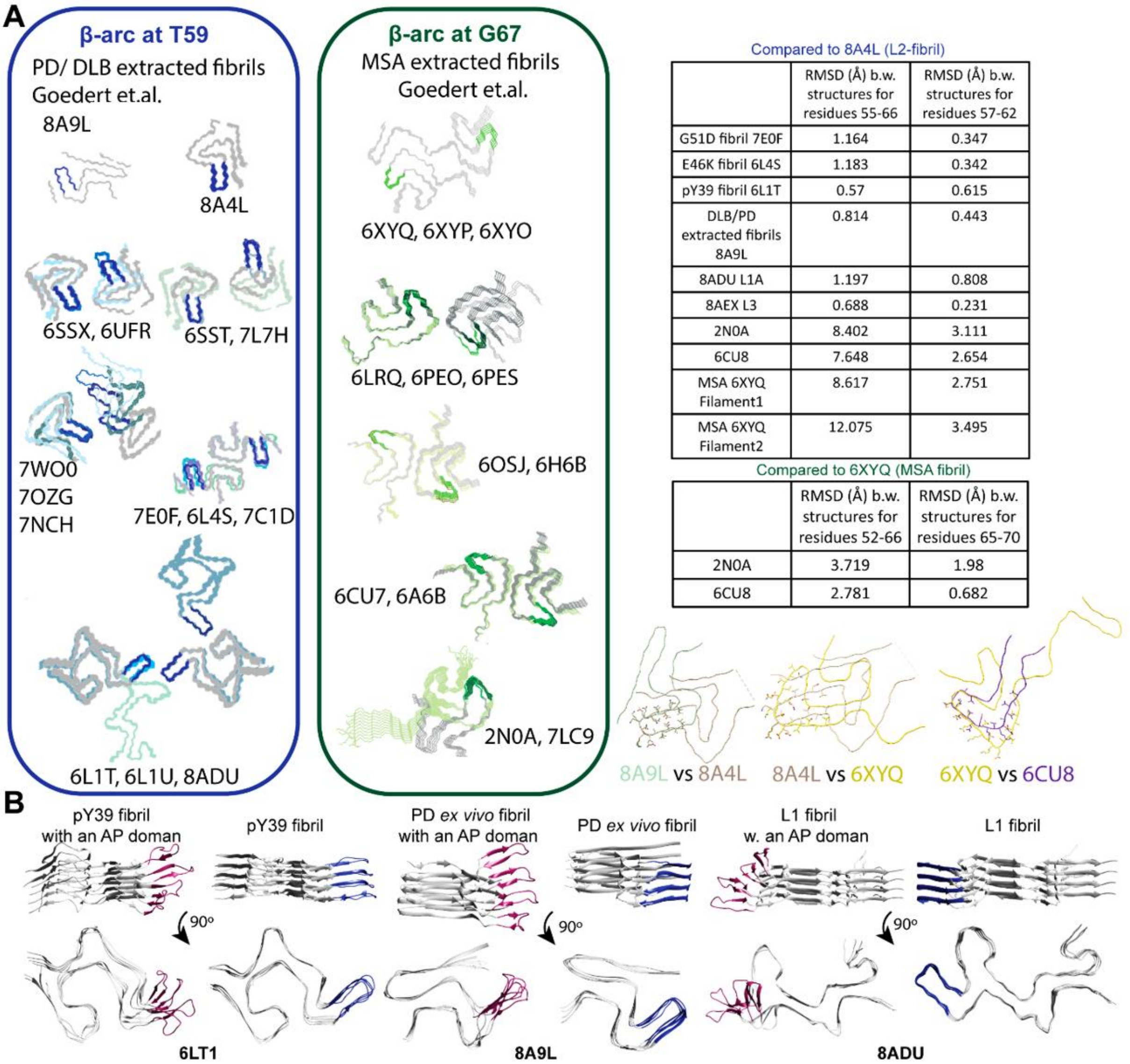

Compared to 8A4L (L2-fibril)

	RMSD (Å) b.w. structures for residues 55-66	RMSD (Å) b.w. structures for residues 57-62
G51D fibril 7E0F	1.164	0.347
E46K fibril 6L4S	1.183	0.342
pY39 fibril 6L1T	0.57	0.615
DLB/PD extracted fibrils 8A9L	0.814	0.443
8ADU L1A	1.197	0.808
8AEX L3	0.688	0.231
2N0A	8.402	3.111
6CU8	7.648	2.654
MSA 6XYQ Filament1	8.617	2.751
MSA 6XYQ Filament2	12.075	3.495

Compared to 6XYQ (MSA fibril)

	RMSD (Å) b.w. structures for residues 52-66	RMSD (Å) b.w. structures for residues 65-70
2N0A	3.719	1.98
6CU8	2.781	0.682

Supplementary Figure.14: Classification of αS fibrils and their respective intermediates. (A) A classification can be made of αS fibrils based on the morphology of the hairpin turn at T59. A β-arc at T59, as found in the L2 fibril type, is conserved in a large set of αS fibril polymorphs (blue), including extracted fibrils from Parkinson's and Lewy Body Dementia patients (8A9L), pY39 recombinant fibril (6L1T), L1 and L3 lipidic polymorphs, E46K (6UFR, 6L4S) and G51D (7E0F) fibrils. By contrast, all other αS fibril polymorphs, like the MSA-fold (6XYQ), has the same type of β-arc at G67 instead (green). This is despite some of these fibrils having a distinct topological fold, for example, Greek-key versus Triple-L. The same distinction has previously been made by Sawaya and Eisenberg et.al. (Cell, 2021)[5] who referred to the β-arc at G67 fibrils as "boot-type" and the β-arc at T59 fibrils as "sandal-type." (B) Snapshots from MD simulations showing different tetrameric oligomers modelled from fibril polymorphs with a β-arc at T59 (blue strands) can accommodate anti-parallel β-strands (pink strands) at this position.

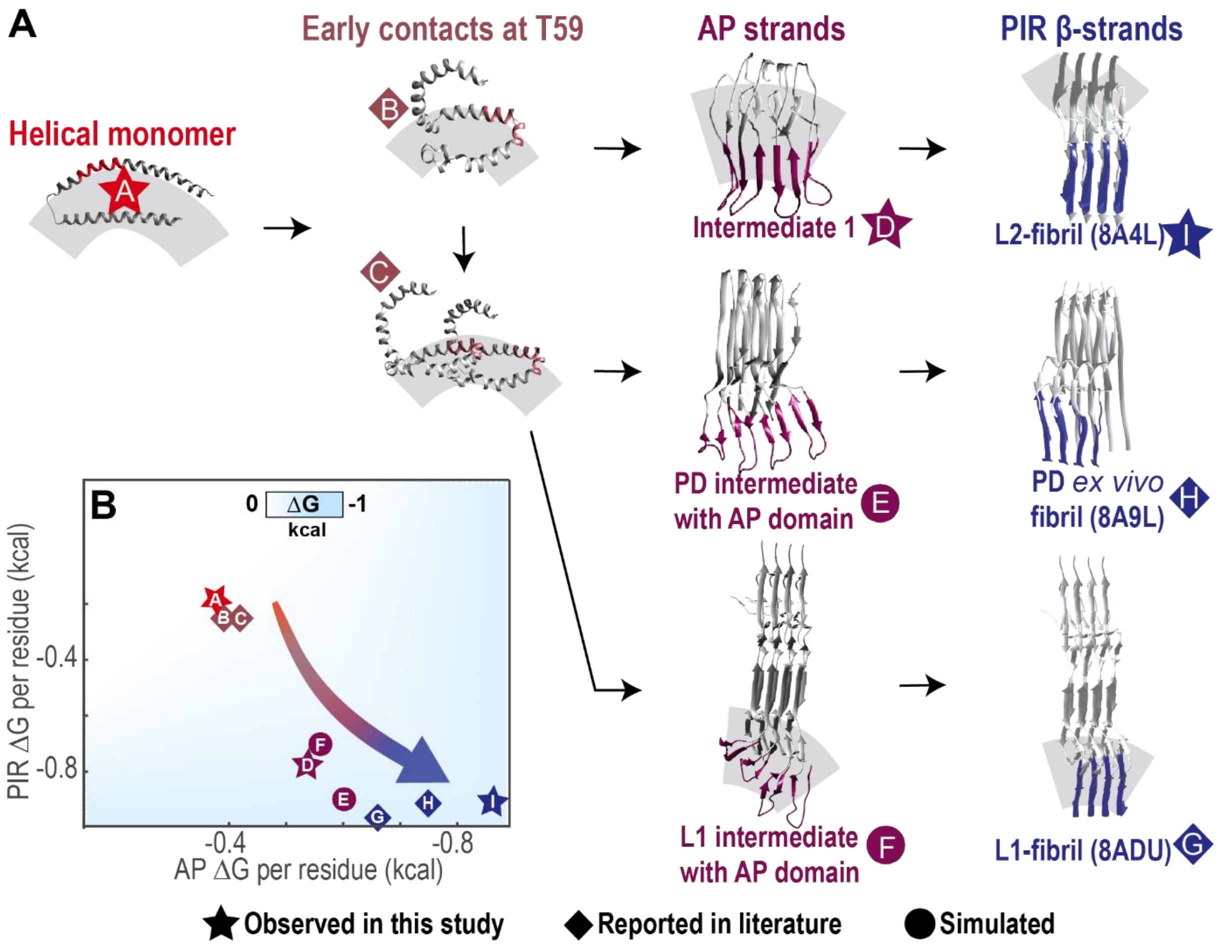

Supplementary Figure.15: Proposed aggregation cascade for αS-fibril polymorphs with a β-arc at T59. (A) Schematic showing transition from the helical αS monomer to PIR fibrils with a β-arc at T59. The residues (V52-V66) that transition from β-hairpin to β-arc are shown with red in the helical state, with light pink when a bend at T59 is introduced, with dark pink when they are structured as AP β-strands and blue in the PIR state. Letters enclosed in a shape represent each structure. The shape determines if the structure was observed in this study (star), observed in literature (diamond) or simulated with all-atom MD (circle). β-hairpins/ AP strands represent an intermediate folding state. The species observed on-pathway to the L2 fibril is referred to as "intermediate 1 (I1)." Intermediates which we propose would have an AP domain like I1 but the rest of the structure is more like their respective fibrils' folds are referred to as "L1 intermediate with AP domain" and "PD intermediate with AP domain". **(B)** Solvation free energy per residue calculations[5,6] for the PIR (y-axis) and AP(x-axis) domains show how an αS molecule is stabilized during the folding of the L2 fibril, L1-fibril, and PD *ex vivo* fibril. The solvation energy does not consider the additional stabilization provided by lipids in lipidic aggregates. Rather, it is an estimate of the intrinsic properties of the structure that contribute to its energetic stability in an aqueous environment. Source data are provided as a Source Data file. **Not only can the structures of L1 and PD *ex* vivo fibrils accommodate AP domains at T59, but the formation of these domains is energetically favorable. Helical monomer (Structure A):** αS starts as a lipid bound helical monomer in the preparation used in this work[1] with relatively high energies for the PIR and AP residues. **Early contacts at T59 (Structures B, C):** A monomer and dimer have been observed that are bent at T59 when bound to lipids[7], which appears to stabilize PIR and AP domains to a small extent. **β-hairpin at T59 (Structures D-F):** To minimize energy in both segments, the PIR residues adopt a fibril like structure, and intra- and inter- molecular H-bond formation is completed in the AP domain in four copies of

αS. This is observed in I1 and simulated in the L1 and PD *ex vivo* fibril structures. **β-arc at T59 (Structures G-I):** Finally, additional stabilization is provided to both, the PIR and AP segments, by the transition to a β-arc at T59. This transition appears to relieve strain in the PIR region due to twist induced by AP strands, reducing the energy per residue for V37-S42 and T75-K80. The β-arc at T59 packs more hydrophobic residues in the fibril core than the AP-strand arrangement, drastically reducing the solvation energy for V52-V66. The relative stability of the fibrils depends on the distinct arrangements of the PIR domains.

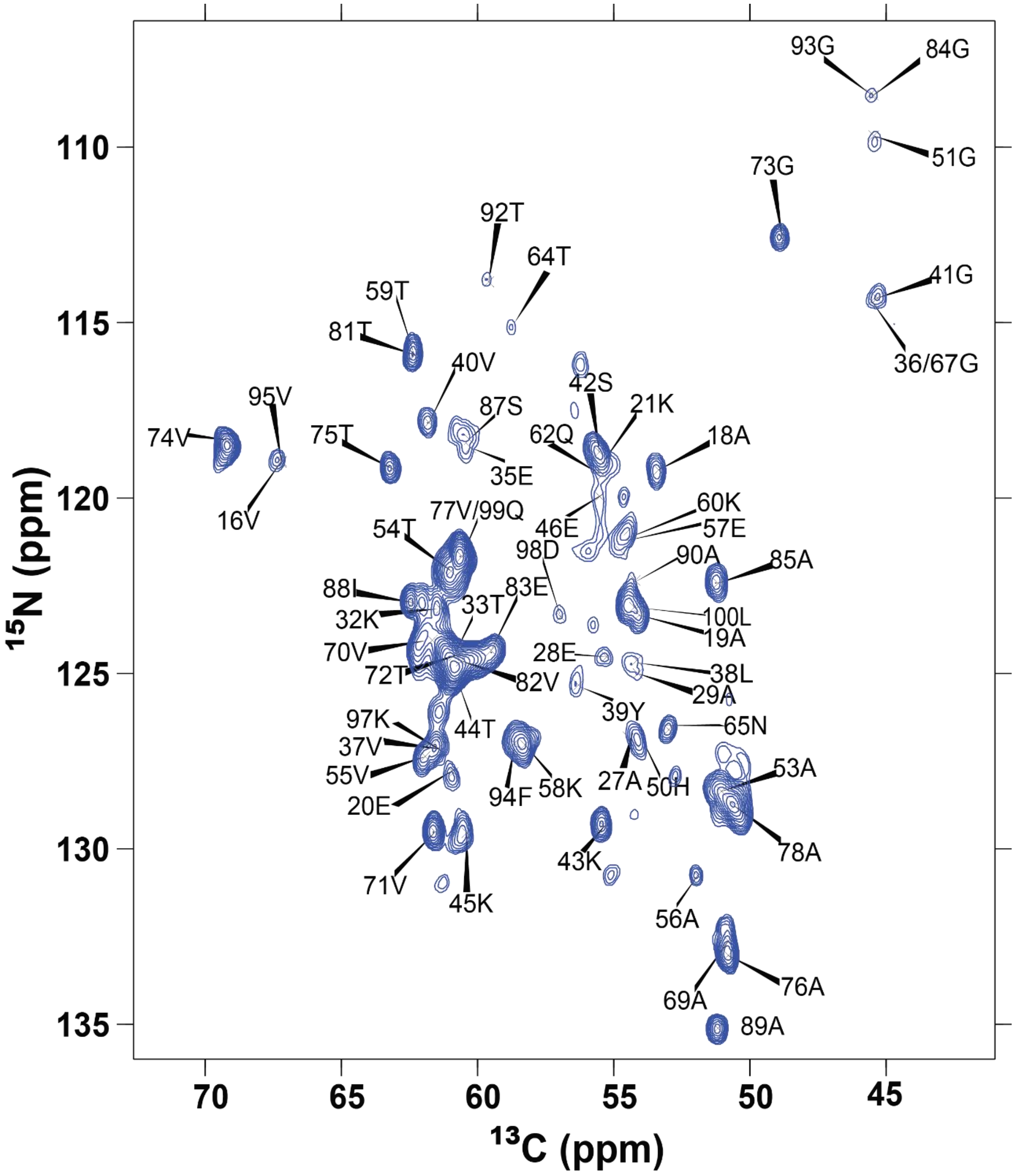

Supplementary Figure 16: Assigned spectra of the structured segments of I1. 2D Cα-N projection from a 3D (H)CANH spectrum with specific resonance assignments. Continuous assignments were obtained for stretches 16-22, 27-32, 34-68 and 69-100, resulting in 64% sequence coverage of what is considered the structured core of αS (residues 1-100).

Supplementary Table.1: Contacts used for CYANA and MD structure calculation

Residues	Restraint	Upper Limit (Å)	Lower Limit (Å)
37-43, 76-80	Nearest-neighbor intermolecular H-bonds between backbone N/H and O of residue i with backbone O and N/H of residues i-1 and i+1, respectively	O-N 3 Å, O-H 2.8 Å	O-N 2.7 Å, O-H 1.8 Å
63 O- 55 N/H, 63 N/H- 55 O	Intra-molecular anti-parallel H-bonds	O-N 3 Å, O-H 2.8 Å	O-N 2.7 Å, O-H 1.8 Å
65 O- 53 N/H, 65 N/H- 53 O	Intra-molecular anti-parallel H-bonds	O-N 3 Å, O-H 2.8 Å	O-N 2.7 Å, O-H 1.8 Å
66 O- 52 N/H, 66 N/H-52 O	Inter-molecular anti-parallel H-bonds	O-N 3 Å, O-H 2.8 Å	O-N 2.7 Å, O-H 1.8 Å
54 O- 64 N/H, 54 N/H-64 O	Inter-molecular anti-parallel H-bonds	O-N 3 Å, O-H 2.8 Å	O-N 2.7 Å, O-H 1.8 Å
	**On account of chemical shift similarity w. L2 fibril**		
	76 Cβ- 71 Cγ	7	
	44 Cγ – 74 Cγ	4	
	47 Cα – 74 Cγ	5	
	77 Cγ- 38 Cδ	7	
	**Observed in (H)NHH and (H)CHH spectra**		
	65 Hδ- 53 H	7	
	62 Hε- 55 H	7	
	54 Hβ – 63 Hγ	7	
	71 Hα-78 H	7.5	
	73 H- 75 Hα	7	
	40 Hα- 77 Hα	7	
	28 Hα- 96 Hα	7	
	30 H_N- 96 H_N	7	
	**Observed in (H)N(H)(H)NH spectra at 3.46 ms mixing**		
	H_N- H_N contacts between A89-T92, G86-K97, V82-T92, G86-I88, T81- S87, K97- V95	4	

Supplementary Table.2: Acquisition parameters for NMR measurements

Spectrum	Transfer	Nucleus	rf (kHz)	time CP (ms)	Ramp	t1 (ms)	t2 (ms)	t3 (ms)	SW3 (ppm)	SW2 (ppm)	SW1 (ppm)	No. of scans	Time (hr)	Temperature Set (K)	Probes/ Magnet	Recyle delay (s)	MAS (kHz)
hNH	1H-15N	1H	105	0.9	80-100 (1H)	20	42.5	-	30	-	36	32	3.5	235	1.3mm/ 800 MHz	1.6	55
		15N	38														
	15N-1H	1H	98	0.55	100-80 (1H)												
		15N	38														
hCaNH	1H-13C	1H	105	1.9	80-100 (1H)	15	6	18.8	34	36	36	8	56				
		13C	41														
	13C-15N	13C	26	13	63-94 (15N)												
		15N	37														
	15N-1H	1H	98	0.35	100-80 (1H)												
		15N	38														
hCoNH	1H-13C	1H	106	4	80-100 (1H)	15	12.8	21.2	30	12	36	8	24				
		13C	41														
	13C-15N	13C	25	10	63-94 (15N)												
		15N	37														
	15N-1H	1H	98	0.35	100-80 (1H)												
		15N	38														
hcoCacoNH	1H-13C	1H	106	4	80-100 (1H)	15	6	18.8	34	36	36	8	150				
		13C	41														
	13C-15N	13C	25	10	63-94 (15N)												
		15N	37														
	15N-1H	1H	98	0.35	100-80 (1H)												
		15N	38														
hCocaNH	1H-13C	1H	106	4	80-100 (1H)	15	15.3	21.2	30	12	36	8	216				
		13C	41														
	13C-15N	13C	26	15	63-94 (15N)												
		15N	37														
	15N-1H	1H	98	0.35	100-80 (1H)												
		15N	38														
hcaCbcaNH	1H-13C	1H	105	1.9	80-100 (1H)	15	5	18.8	34	74	36	4	405				
		13C	41														
	13C-15N	13C	26	15	63-94 (15N)												
		15N	37														
	15N-1H	1H	98	0.33	100-80 (1H)												
		15N	38														
H(h)NH	1H-15N	1H	105	0.9	80-100 (1H)	15	3	42.5	30	14	35	8	240				
		15N	38														
	15N-1H	1H	98	0.55													
		15N	38		100-80 (1H)												
	13C 90°		63														
	1H 90°		111														
13C-13C DARR	1H-13C	1H	82	1.5		8.1	8.6	-	230	275	-	8	99	265	3.2mm/ 850 MHz	1.8	17
		13C	46														
		DARR (ms)	20														
hNhhNH	1H-15N	1H	105	0.95	80-100 (1H)	9	9	21	38	38	40	16	192	238	1.3 mm/ 1200 MHz	0.6	55.555
		15N	33														
	15N-1H	1H	100	0.5	100-80 (1H)												
		15N	33														

Supplementary Table.3: Overview of MD simulation systems and setup for runs with distance restraints.

System	Total no. simulations	Box dimensions	Total no. of atoms	Total no. of water molecules	Salt	Lipid composition	Figure(s)
8a4l w/o morph (G36-K97)	3 x L2 fibril AP morphology	10.7 x 10.7 x 7.6 nm	87993	28089	150 mM NaCl	n/a	S13
8a4l to I1 morph (G36-T81)	3 x open; 3 x bowl; 3 x barrel AP morphology	10.7 x 10.7 x 7.6 nm	89113	28768	150 mM NaCl	n/a	S13, S14
8a4l to I1 morph (V16-Q99)	2 x open; 2 x bowl AP morphology	12.6 x 12.6 x 8.9 nm	143404	46108	150 mM NaCl	n/a	S12
8a4l to I1 morph with additional structure restraints<br>(V16-Q99)	3 x open; 3 x bowl AP morphology	12.6 x 12.6 x 8.9 nm	143404	46108	150 mM NaCl	n/a	
8adu to I1 morph (M1-Q99)	1 x open AP morphology	13.9 x 13.9 x 9.8 nm	192612	62172	150 mM NaCl	n/a	S13
8a9l to I1 morph (G31-L100)	1 x open AP morphology	11.3 x 11.3 x 7.9 nm	102291	32675	150 mM NaCl	n/a	S13
6l1t to I1 morph (M1-Q99)	1 x open AP morphology	13.0 x 13.0 x 9.2 nm	157839	50601	150 mM NaCl	n/a	S13
I1 Orientation 1 (G36-T81)	3 x open AP morphology	8.5 x 8.2 x 9.2 nm	64594	13634	150 mM NaCl	POPC: 79; POPA: 88	S8
I1 Orientation 2 (G36-T81)	3 x open AP morphology	8.6 x 8.3 x 9.2 nm	67702	13281	150 mM NaCl	POPC: 97; POPA: 103	S8
I1 Orientation 3 (G36-T81)	3 x open AP morphology	8.9 x 8.5 x 8.9 nm	69823	12716	150 mM NaCl	POPC: 115; POPA: 115	S8
I1 Orientation 4 (G36-T81)	3 x open AP morphology	8.5 x 8.2 x 9.1 nm	65846	13638	150 mM NaCl	POPC: 89; POPA: 85	S8
I1 Orientation 5 (G36-T81)	3 x open AP morphology	8.8 x 8.4 x 8.9 nm	68397	13256	150 mM NaCl	POPC: 105; POPA: 101	S8
I1 Orientation 6 (G36-T81)	3 x open AP morphology	8.7 x 8.4 x 9.1 nm	68576	12942	150 mM NaCl	POPC: 105; POPA: 110	S8
I1 Orientation 7 (G36-T81)	3 x open AP morphology	8.7 x 8.4 x 9.4 nm	70505	12525	150 mM NaCl	POPC: 120; POPA: 120	S8
I1 Orientation 8 (G36-T81)	3 x open AP morphology	8.8 x 8.5 x 8.8 nm	68036	13325	150 mM NaCl	POPC: 102; POPA: 99	S8

Supplementary Table.4: Overview of MD simulation systems and setup for runs with distance restraints.

System	Total no. simulations	Box dimensions	Total no. of atoms	Total no. of water molecules	Salt	Lipid composition	Figure(s)
I1 Orientation 1 (G36-T81)	3 x open; 3 x bowl; 3 x barrel AP morphology	8.5 x 8.2 x 9.2 nm	64594	13634	150 mM NaCl	POPC: 79; POPA: 88	S8
I1 Orientation 2 (G36-T81)	3 x open; 3 x bowl; 3 x barrel AP morphology	8.6 x 8.3 x 9.2 nm	67702	13281	150 mM NaCl	POPC: 97; POPA: 103	S8
I1 Orientation 3 (G36-T81)	3 x open; 3 x bowl; 3 x barrel AP morphology	8.9 x 8.5 x 8.9 nm	69823	12716	150 mM NaCl	POPC: 115; POPA: 115	S8
I1 Orientation 4 (G36-T81)	3 x open; 3 x bowl; 3 x barrel AP morphology	8.5 x 8.2 x 9.1 nm	65846	13638	150 mM NaCl	POPC: 89; POPA: 85	S8
I1 Orientation 1 (V16-Q99)	6 x open; 3 x bowl; 3 x barrel AP morphology	13.8 x 13.3 x 12.8 nm	227726	54971	150 mM NaCl	POPC: 230; POPA: 229	4, S9
I1 Orientation 2 (V16-Q99)	3 x open; 3 x bowl; 3 x barrel AP morphology	13.2 x 12.7 x 13.4 nm	230169	54614	150 mM NaCl	POPC: 244; POPA: 243	4, S9
I1 Orientation 3 (V16-Q99)	3 x open; 3 x bowl; 3 x barrel AP morphology	13.2 x 12.7 x 13.3 nm	230202	54335	150 mM NaCl	POPC: 247; POPA: 247	S9
I1 Orientation 1 with additional structure restraints (V16-Q99)	3 x open; 3 x bowl AP morphology	13.9 x 13.4 x 12.4 nm	217544	51736	150 mM NaCl	POPC: 229; POPA: 226	S10, S11
I1 Orientation 2 with additional structure restraints (V16-Q99)	3 x open; 3 x bowl AP morphology	13.9 x 13.4 x 12.4 nm	220622	51234	150 mM NaCl	POPC: 244; POPA: 248	S10, S11
I1 Orientation 1 with additional structure restraints (V16-Q99)	5 x open; 5 x bowl AP morphology	13.9 x 13.4 x 12.4 nm	217490	51709	100 mM NaCl, 40 mM $CaCl_2$	POPC: 229; POPA: 226	S10, S11
I1 Orientation 2 with additional structure restraints (V16-Q99)	5 x open; 5 x bowl AP morphology	13.9 x 13.4 x 12.4 nm	220568	51207	100 mM NaCl, 40 mM $CaCl_2$	POPC: 244; POPA: 248	S10, S11

References for Supplementary Information

1. Antonschmidt, L. *et al.* Insights into the molecular mechanism of amyloid filament formation: Segmental folding of α-synuclein on lipid membranes. *Sci Adv* 7, eabg2174 (2021).

2. Comellas, G., Lemkau, L. R., Zhou, D. H., George, J. M. & Rienstra, C. M. Structural intermediates during α-synuclein fibrillogenesis on phospholipid vesicles. *J Am Chem Soc* 134, 5090–5099 (2012).

3. Frieg, B. *et al.* The 3D structure of lipidic fibrils of α-synuclein. *Nature Communications 2022 13:1* 13, 1–10 (2022).

4. Weinreb, P. H., Zhen, W., Poon, A. W., Conway, K. A. & Lansbury, P. T. NACP, A Protein Implicated in Alzheimer's Disease and Learning, Is Natively Unfolded. *Biochemistry* 35, 13709–13715 (1996).

5. Sawaya, M. R., Hughes, M. P., Rodriguez, J. A., Riek, R. & Eisenberg, D. S. The expanding amyloid family: Structure, stability, function, and pathogenesis. *Cell* 184, 4857–4873 (2021).

6. Eisenberg, D. & Mclachlan, A. D. Solvation energy in protein folding and binding. *Nature* 319, 199–203 (1986).

7. Schwarz, T. C. *et al.* High-resolution structural information of membrane-bound α-synuclein provides insight into the MoA of the anti-Parkinson drug UCB0599. *Proc Natl Acad Sci U S A* 120, e2201910120 (2023).
