# Supplementary material for: Lipidic folding pathway of α-Synuclein via a toxic oligomer
Source: Nat Commun. 2025 Jan 17;16:760. doi: 10.1038/s41467-025-55849-3 (PMC11742675; doi:10.1038/s41467-025-55849-3)
Supplement: Supplementary file 2 — Description of Additional Supplementary Files [file 41467_2025_55849_MOESM2_ESM.pdf]

Description of Additional Supplementary Files

File Name: Supplementary Movie 1
Description: Transition from β-hairpin to β-arc. A trajectory is mapped between an I1 conformer and the L2-fibril. The AP domain residues V52-V66 are highlighted in pink when they form a β-hairpin, as in I1. The H-bonds between the two strands in the AP domain are slowly seen breaking as the segment transitions to an L2-fibril type β-arc which is represented in blue. The video plays from the β-arc back to the β-hairpin with a 90˚rotation.

File Name: Supplementary Movie 2
Description: Snapshots from the unrestrained MD simulation of the I1 open morphology in orientation 1 in the bilayer. In this orientation, the PIR and AP domains are in the same leaflet of the lipid bilayer. Pink ribbons represent the AP domain, green ribbons the PIR domain. Blue spheres represent the POPC headgroup nitrogen and a surface map of lipids is shown. During the course of the simulation headgroups are pulled toward charged residues located in the hydrophobic core of the bilayer.

File Name: Supplementary Movie 3
Description: Snapshots from the unrestrained MD simulation of the I1 open morphology in orientation 2 in the bilayer. In this orientation, the PIR and AP domains are in different leaflets of the bilayer. Pink ribbons represent the AP domain, green ribbons the PIR domain. Blue spheres represent the POPC headgroup nitrogen and a surface map of lipids is shown. During the course of the simulation headgroups are pulled toward charged residues located in the hydrophobic core of the bilayer.

File Name: Supplementary Data 1
Description: Code to identify single-molecules and extract the intensity time traces in the photobleaching experiment was done using custom written MATLAB code.
