# Supplementary material for: Lipidic folding pathway of α-Synuclein via a toxic oligomer
Source: Nat Commun. 2025 Jan 17;16:760. doi: 10.1038/s41467-025-55849-3 (PMC11742675; doi:10.1038/s41467-025-55849-3)
Supplement: Supplementary file 7 — Reporting Summary [file 41467_2025_55849_MOESM7_ESM.pdf]

nature portfolio

Corresponding author(s): B. L. de Groot
C. Griesinger
L. Andreas

Last updated by author(s): Nov 27, 2024

Reporting Summary

Nature Portfolio wishes to improve the reproducibility of the work that we publish. This form provides structure for consistency and transparency in reporting. For further information on Nature Portfolio policies, see our Editorial Policies and the Editorial Policy Checklist.

Statistics

For all statistical analyses, confirm that the following items are present in the figure legend, table legend, main text, or Methods section.

n/a	Confirmed	
☐	☒	The exact sample size (n) for each experimental group/condition, given as a discrete number and unit of measurement
☐	☒	A statement on whether measurements were taken from distinct samples or whether the same sample was measured repeatedly
☐	☒	The statistical test(s) used AND whether they are one- or two-sided<br>*Only common tests should be described solely by name; describe more complex techniques in the Methods section.*
☒	☐	A description of all covariates tested
☒	☐	A description of any assumptions or corrections, such as tests of normality and adjustment for multiple comparisons
☐	☒	A full description of the statistical parameters including central tendency (e.g. means) or other basic estimates (e.g. regression coefficient) AND variation (e.g. standard deviation) or associated estimates of uncertainty (e.g. confidence intervals)
☐	☒	For null hypothesis testing, the test statistic (e.g. F, t, r) with confidence intervals, effect sizes, degrees of freedom and P value noted<br>*Give P values as exact values whenever suitable.*
☒	☐	For Bayesian analysis, information on the choice of priors and Markov chain Monte Carlo settings
☒	☐	For hierarchical and complex designs, identification of the appropriate level for tests and full reporting of outcomes
☒	☐	Estimates of effect sizes (e.g. Cohen's d, Pearson's r), indicating how they were calculated

Our web collection on statistics for biologists contains articles on many of the points above.

Software and code

Policy information about availability of computer code

Data collection	NMR Experiments: Bruker Topspin<br>MD simulations: GROMACS 2022 (including implementations of P-LINCS, SETTLE, non-bonded Verlet scheme, PME, velocity-rescale Temperature coupling and Parrinello-Rahman barostat); CHARMM36m protein force field; CHARMM36 lipid parameters; CHARMM-modified TIP3P water model;<br>Super-resolution Fluorescence measurements: ANDOR SOLIS imaging software
Data analysis	NMR experiments, Bruker Topspin (4.0.07), CcpNMR (2.4.2), NMRFAM-Sparky (3.1.9), CYANA (3.98.15)<br>For the analysis of the MD simulation trajectories, the following software and tools were used:<br>GROMACS version 2022 (https://www.gromacs.org/): gmx hbond, gmx mindist;<br>GROMACS version 2022 (https://www.gromacs.org/): gmx hbond, gmx mindist; gmx denisty; gmx gyrate<br>g_contacts (Blau et.al.) used to calculate interatomic distances;<br>Fortran code was used to obtain hydrogen bond energies (Espinosa et. al.)<br>For rendering and plotting, the following software was used: ChimeraX, (1.8) gnuplot 5.4, seaborn, matplotlib and pyplot libraries from Python 3.7.<br><br>Super-resolution Fluorescence miscroscopy: DISC algorithm (White et.al.) MATLAB scripts were used to analyze raw image stacks.]. The code to identify single-molecules and extract the intensity time traces in the photobleaching experiment was done using custom written MATLAB code. However, any published codes such as ThunderSTORM can be easily used for the purpose, and available from Ovesny et.al.. The code to fit the intensity time traces is available from White et.al.. The code to fit the binomial distribution of the number of dyes per aggregate was written in MATLAB and provided as a source data file “Binomfit.txt”. The code to analyze the polarCOLD data was written by a previous lab

member and described in the following Böning et.al.

For manuscripts utilizing custom algorithms or software that are central to the research but not yet described in published literature, software must be made available to editors and reviewers. We strongly encourage code deposition in a community repository (e.g. GitHub). See the Nature Portfolio guidelines for submitting code & software for further information.

Data

Policy information about availability of data

All manuscripts must include a data availability statement. This statement should provide the following information, where applicable:

- Accession codes, unique identifiers, or web links for publicly available datasets
- A description of any restrictions on data availability
- For clinical datasets or third party data, please ensure that the statement adheres to our policy

Assigned chemical shift data for αS Intermediate 1 were deposited in the BMRB under the accession number 52283. Tables used for structure determination are provided in the Supplement. Source data will be provided with this paper as Source Data. MD simulation data and parameter files are provided through the Edmond data repository at [https://doi.org/10.17617/3.0V1ODV]. NMR spectra are deposited at Edmond under [https://doi.org/10.17617/3.TXND2C].

Research involving human participants, their data, or biological material

Policy information about studies with human participants or human data. See also policy information about sex, gender (identity/presentation), and sexual orientation and race, ethnicity and racism.

Reporting on sex and gender	N/A
Reporting on race, ethnicity, or other socially relevant groupings	N/A
Population characteristics	N/A
Recruitment	N/A
Ethics oversight	N/A

Note that full information on the approval of the study protocol must also be provided in the manuscript.

Field-specific reporting

Please select the one below that is the best fit for your research. If you are not sure, read the appropriate sections before making your selection.

☒ Life sciences ☐ Behavioural & social sciences ☐ Ecological, evolutionary & environmental sciences

For a reference copy of the document with all sections, see nature.com/documents/nr-reporting-summary-flat.pdf

Life sciences study design

All studies must disclose on these points even when the disclosure is negative.

Sample size	NMR experiments: Number of time points in the direct and indirect dimensions were adapted for resolution and singal to noise optimization. Samples tested were transient and multiple samples had to be prepared to obtain assignment spectra and contact spectra. The transient nature of the sample was monitored as described in the Supplement and methods. Once the sample began to change, a new one was prepared for the next measurement. With this methodology, an Intermediate 1 sample was found to be repeatable. Sample sizes and repetitions were determined based on signal to noise ratio required for each spectrum and sample yield.<br>MD simulations: a total of 22 MD simulations of embedded structures were run for 100 ns with distance restraints and an additional 500 ns without restraints to collect data that are evaluated against experimental measurements. For details on sample size and simulation length please refer to MD checklist.<br>Cell experiments were repeated for 6 replicates, often from two separate preparations. Sample size was chosen based on standard practices in the field (i.e. 3 or more biological replicates).
Data exclusions	no data were excluded
Replication	NMR measurements: For each sample prepared, a finger spectrum was obtained that confirmed robust reproducibility of the preparation.<br>MD simulations: in all, for different morphologies and poses, 62 simulations were run. Each condition was run atleast in triplicates.
Randomization	no randomization was performed.
Blinding	no blinding was performed

Reporting for specific materials, systems and methods

We require information from authors about some types of materials, experimental systems and methods used in many studies. Here, indicate whether each material, system or method listed is relevant to your study. If you are not sure if a list item applies to your research, read the appropriate section before selecting a response.

Materials & experimental systems

n/a	Involved in the study
☒	☐ Antibodies
☐	☒ Eukaryotic cell lines
☒	☐ Palaeontology and archaeology
☒	☐ Animals and other organisms
☒	☐ Clinical data
☒	☐ Dual use research of concern
☒	☐ Plants

Methods

n/a	Involved in the study
☒	☐ ChIP-seq
☒	☐ Flow cytometry
☒	☐ MRI-based neuroimaging

Eukaryotic cell lines

Policy information about cell lines and Sex and Gender in Research

Cell line source(s)	SH-SY5Y cells were obtained from ATCC (CRL-2266).
Authentication	SH-SY5Y cells were authenticated by short tandem repeat profiling by ATCC. AT our end, authentication was performed by morphology and replication rate.
Mycoplasma contamination	No mycoplasma was detected in the cultures.
Commonly misidentified lines (See ICLAC register)	No commonly misidentified cell lines were used.

Plants

Seed stocks	N/A
Novel plant genotypes	N/A
Authentication	N/A
